# High-throughput screening of the *Saccharomyces cerevisiae* genome for 2-amino-3-methylimidazo [4,5-*f*] quinoline resistance identifies colon cancer-associated genes

**DOI:** 10.1093/g3journal/jkad219

**Published:** 2023-09-22

**Authors:** Michael Dolan, Nick St. John, Faizan Zaidi, Francis Doyle, Michael Fasullo

**Affiliations:** College of Nanotechnology, Science, and Engineering, State University of NewYork at Albany, Albany, NY 12203, USA; College of Nanotechnology, Science, and Engineering, State University of NewYork at Albany, Albany, NY 12203, USA; College of Nanotechnology, Science, and Engineering, State University of NewYork at Albany, Albany, NY 12203, USA; College of Nanotechnology, Science, and Engineering, State University of NewYork at Albany, Albany, NY 12203, USA; College of Nanotechnology, Science, and Engineering, State University of NewYork at Albany, Albany, NY 12203, USA

**Keywords:** genome profiling, budding yeast, heterocyclic aromatic amine, colon cancer

## Abstract

Heterocyclic aromatic amines (HAAs) are potent carcinogenic agents found in charred meats and cigarette smoke. However, few eukaryotic resistance genes have been identified. We used *Saccharomyces cerevisiae* (budding yeast) to identify genes that confer resistance to 2-amino-3-methylimidazo[4,5-*f*] quinoline (IQ). CYP1A2 and NAT2 activate IQ to become a mutagenic nitrenium compound. Deletion libraries expressing human CYP1A2 and NAT2 or no human genes were exposed to either 400 or 800 µM IQ for 5 or 10 generations. DNA barcodes were sequenced using the Illumina HiSeq 2500 platform and statistical significance was determined for exactly matched barcodes. We identified 424 ORFs, including 337 genes of known function, in duplicate screens of the “humanized” collection for IQ resistance; resistance was further validated for a select group of 51 genes by growth curves, competitive growth, or trypan blue assays. Screens of the library not expressing human genes identified 143 ORFs conferring resistance to IQ per se. Ribosomal protein and protein modification genes were identified as IQ resistance genes in both the original and “humanized” libraries, while nitrogen metabolism, DNA repair, and growth control genes were also prominent in the “humanized” library. Protein complexes identified included the casein kinase 2 (CK2) and histone chaperone (HIR) complex. Among DNA Repair and checkpoint genes, we identified those that function in postreplication repair (*RAD18*, *UBC13*, *REV7*), base excision repair (*NTG1*), and checkpoint signaling (*CHK1*, *PSY2*). These studies underscore the role of ribosomal protein genes in conferring IQ resistance, and illuminate DNA repair pathways for conferring resistance to activated IQ.

## Introduction

The heterocyclic aromatic amine (HAA) 2-amino-3-methylimidazo[4,5-*f*] quinoline (IQ) is a mutagen (Kasai et al. 1980) and a class 2A carcinogen (IARC 1993; Olalekan Adeyeye and Ashaolu 2021), present in charred meat and cigarette smoke. IQ and other heterocyclic aromatic amines (HAAs) are generated by Maillard reactions involving creatine, sugar, and an amino acid (Jägerstad et al. 1991); these reactions occur when charring meat and may suggest why red meat consumption increases colon cancer risk (Sinha et al. 1999; Sinha et al. 2001; Linseisen et al. 2002; Aykan 2015; Gongora et al. 2019). While charred meats contain a mixture of HAAs, IQ exposure has been directly correlated to inducing cancer. For example, IQ exposure induces liver, kidney, and colon cancers in rats (Turesky and Markovic 1995). Although the exact mechanism by which IQ induces cancer is unknown, IQ exposure is associated with oxidative stress (Carvalho et al. 2015) and higher levels of DNA damage (de Carvalho et al. 2016). Genotoxic endpoints of IQ exposure include higher frequencies of both mutations (Aeschbacher and Turesky 1991) and sister chromatid exchange (Sawada et al. 1994). These studies suggest that IQ acts as a genotoxin contributing to the carcinogenicity associated with red meat or cigarette smoke.

**Fig. 1.**
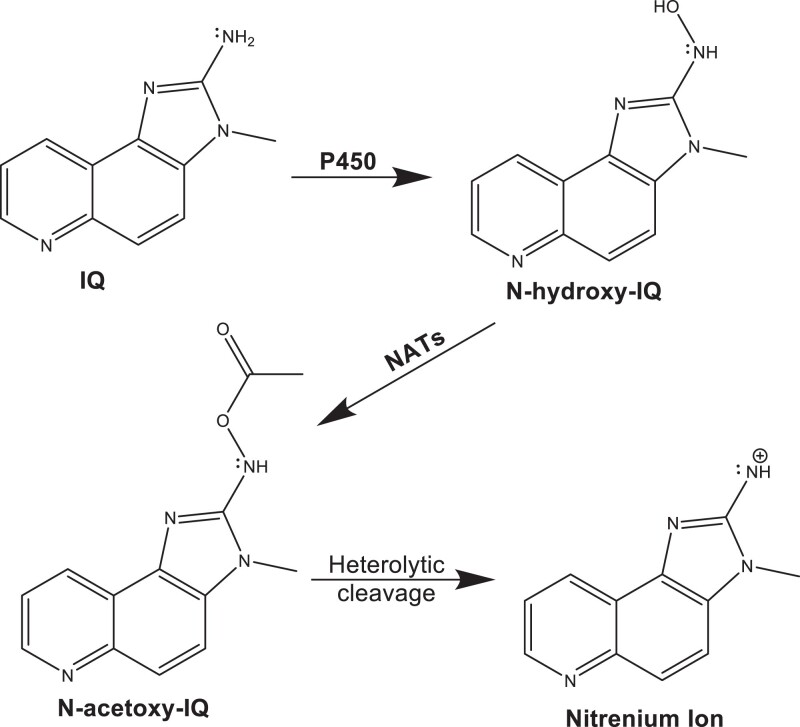
Bioactivation of the HAA IQ by human CYP and NAT proteins. The chemical structures of IQ, N-hydroxy IQ, and N-acetoxy-IQ are shown in the figure. IQ is first hydroxylated by CYPs to form N-hydroxy-IQ. The hydroxylated IQ is then acetylated by N-acetyl transferases (NATs) to form N-acetyl-IQ. This compound is unstable and generates the nitrenium ion shown in the figure (for review see, Turesky and Le Marchand 2011).

IQ pre-incubated with rat liver S9 is more mutagenic in the Ames assay than IQ alone (Kasai et al. 1980), indicating that IQ's genotoxicity requires bioactivation. In humans, bioactivation occurs by a 2-step mechanism (Fig. 1; Kim and Guengerich 2005). First, cytochrome P450 enzymes (CYPs), such as CYP1A2, hydroxylate IQ to form N-hydroxy-IQ, and second, N-acetyltransferases (NATs), such as NAT1 and NAT2, acetylate the N-hydroxy-IQ to form N-acetoxy-IQ (Fig. 1). This latter compound is unstable and undergoes spontaneous heterolytic cleavage to generate a highly reactive nitrenium ion (Kato 1986; Turesky and Vouros 2004; Kim and Guengerich 2005), which forms C^8^ and N^2^ Gua adducts. Interestingly, the N^2^ Gua adduct appears refractory to nucleotide excision repair (NER) and is bypassed by error-prone polymerase (Stavros et al. 2014), while the C^8^ adduct is more efficiently removed (Turesky et al. 1996). NAT2 expression increases the mutagenicity of IQ in both CYP1A2-expressing strains of *Salmonella typhimurium* and in Chinese hamster ovary cells (CHO) (Grant et al. 1992; Josephy et al. 1995) and the recombinogenicity of IQ in CYP1A2-expressing strains of *Saccharomyces cerevisiae* (budding yeast, Paladino et al 1999). These studies indicate that expression of both cytochrome P450 and NAT enzymes are required for high levels of IQ genotoxicity.

Genetic factors that increase the mutagenicity of HAAs have been correlated to higher incidence of smoking and diet-associated cancers. For example, the human NAT2*4 rapid acetylator allele, whose expression in CHO cells increases HAA-associated mutations more than that of *NAT2* slow acetylator alleles (Probst-Hensch et al. 1995; Grant et al. 1997; Fretland et al. 2001), is a colon cancer risk factor among Caucasians (Butler et al. 2008) and smokers (Slattery et al. 1998; Nöthlings et al. 2009). Individuals who frequently consume red meat and exhibit rapid acetylator NAT phenotypes, carrying NAT2*4 or NAT1*10 alleles, are at a higher risk for developing colon cancer (Lilla et al. 2006; Metry et al. 2010; Wang et al. 2011; Le Marchand 2021). Although HAAs are found in varying concentrations in the diet and cigarette smoke, the epidemiological studies suggest that HAA genotoxicity aggravates cancer risk.

Besides DNA adducts, IQ exposure also generates DNA strand breaks and oxidative stress, suggesting that DNA repair genes may function in suppressing IQ-associated genotoxicity (Pfau et al. 1999; Pezdirc et al. 2013). The importance of this question is underscored by observations that high-risk factors for colon cancer include biallelic mutations of 2 base excision repair genes, *NTHL1* (Weren et al. 2015; Te Paske et al. 2020), and *hMYH* (Al-Tassan et al 2002; Wooden et al. 2004). Low penetrant colon cancer risk factors include polymorphisms in the DNA repair genes *RAD18, ERCC5*, *XPC, PARP*, and *APE1* (Pan et al. 2012; Aggarwal et al. 2017; Matejcic et al. 2021). Whether DNA repair gene polymorphisms found specifically in colon cancer confer higher levels of HAA genotoxicity is unknown.

Budding yeast is a model organism for profiling genes that confer resistance to genotoxins. Over 30% of the yeast genome is orthologous to humans (O’Brien et al. 2005), and human orthologs can functionally substitute for 469 essential yeast genes (Kachroo et al. 2015). Each nonessential gene has been individually knocked out and tagged with both upstream and downstream barcodes (uptags and downtags, Giaever et al. 2002); this deletion library has already been shown to be a powerful reagent in identifying drug targets and toxicant resistance genes (Jo et al. 2009; Giaever and Nislow 2014; De La Rosa et al. 2017). While budding yeast does not bioactivate HAAs, expression of human CYP1A2 and NAT2 increases the HAA-associated translocation frequencies in budding yeast (Paladino et al. 1999). A “humanized” yeast deletion library expressing CYP1A2 and NAT2 has already been used to profile the yeast genome for aflatoxin B_1_ (AFB_1_) resistance (St. John et al. 2020). In this study, we profiled the same “humanized” deletion collection for resistance to IQ. We chose IQ because activated IQ is the most recombinogenic HAA in yeast (Paladino et al. 1999) and is highly mutagenic in strains of *S. typhimurium* (Knasmüller et al. 1999; Nohmi and Watanabe 2021). We identified budding yeast genes that confer IQ resistance; mammalian orthologues of a subset of the IQ resistance genes are associated risk factors for colon cancer.

## Materials and methods

### Media and chemicals

Standard media were used for the culture of yeast and bacterial strains (Burke et al. 2000). The bacterial DH1 strains containing the vectors pCS316 and pCYP1A2_NAT2 were cultured in LB-Amp (Luria Broth media with 100 µg/mL ampicillin). Media used for the culture of yeast cells included YPD (yeast extract, peptone, dextrose), SC (synthetic complete, dextrose), and SC-URA (SC lacking uracil). A mouse monoclonal antibody to CYP1A2 (ab22717) and a mouse polyclonal antibody to NAT2 (ab88443) were purchased from Abcam. Purified recombinant NAT2 protein was purchased from OriGene (TP761755). A goat antimouse IgG-HRP antibody was purchased from Santa Cruz Biotechnology (sc-2005). 2-amino-3-methylimidazo [4,5-*f*] quinoline (IQ) was purchased from Toronto Research Chemicals. Dimethyl sulfoxide (DMSO), methanol, and glacial acetic acid were purchased from Sigma. AFB1 was dissolved in DMSO, while IQ was dissolved in methanol containing 0.1% acetic acid, and is simply referred to as MeOH.

### Strains and plasmids

Yeast strains used in this study were derived from either BY4741, BY4743 (Brachmann et al. 1998), YA289, or YB198 (Fasullo et al. 2001) and are of the S288C background. Strain genotypes are listed in Supplementary Table 1. The yeast deletion collection is derived from BY4743, whose genotype is *MAT*a*/α his3Δ1/his3Δ1 leu2Δ0/leu2Δ0 LYS2/lys2Δ0 met15Δ0/MET15  ura3Δ0/ura3 Δ0*. The diploid and haploid homozygous deletion libraries were purchased from Open Biosystems and are now available from Horizon Discovery (https://horizondiscovery.com/en/non-mammalian-research-tools/products/yeast-knockout). The pooled diploid homozygous deletion library (*N* = 4607) was a gift of C. Vulpe (University of Florida). The CYP1A2 expression plasmid, pCS316 (Sengstag et al. 1996), was obtained by CsCl centrifugation (Ausubel 2003). The plasmid used in this study, pCYP1A2_NAT2 (St. John et al. 2020), was constructed by removing the human oxidoreductase sequence from pCS316 and replacing it with the Not1 fragment containing human NAT2 from pRS424-CYP1A2_NAT2. The cDNA of NAT2 was previously positioned in the pGAC.24 (Lesser and Guthrie 1993) expression plasmid under the control of the glycerol phosphate dehydrogenase (GPD) promoter.

### Transformation protocol

The modified diploid homozygous deletion libraries were prepared as previously described (St. John et al. 2020). In brief, 5-fluoroorotic acid resistant isolates were taken from each strain in the diploid homozygous deletion collection and inoculated in 100 μL of YPD in 96-well plates. After overnight incubation at 30°C, plates were centrifuged, washed, and resuspended in 1-step buffer (50% PEG, MW 3300, 0.2 N lithium acetate, 100 mM dithiothreitol, 500 mg/mL denatured salmon sperm DNA). Each well received 1 µg of pCYP1A2_NAT2 and plates were incubated for 30 mins at 30°C, before pipetting 10 μL onto duplicate SC-URA plates. Two Ura^+^ transformants were chosen from each strain and frozen in SC-URA with 7.5% DMSO in 96-well plates. This strategy was successful in transforming approximately 90% of the diploid homozygous deletion library.

To determine the retention of pCYP1A2_NAT2 in wild type and diploid mutants, Ura^+^ transformants were selected in SC-URA and then diluted, approximately 10^5^ cells were exposed to no treatment, 2% MeOH, 400 µM IQ or 800 µM IQ, and incubated for 20 hours. An appropriate dilution was then plated on YPD to obtain single colonies. URA^+^ colonies were counted after replica plating onto SC-URA plates and a 2-day incubation.

### Functional profiling of the yeast genome

We profiled the yeast genome for resistance to the HAA IQ using 2 different pooled collections of the diploid nonessential deletion strains, derived from BY4743. The first pool was a yeast deletion collection library of 4607 knock-out strains, each strain containing a single deletion in a nonessential gene (Jo et al. 2009). The “humanized” pool expressed CYP1A2 and NAT2 in approximately 4,900 strains derived from the 5,417 diploid strains (St. John et al. 2020).

Tubes of the pooled library (10 plates per tube) were thawed on ice and 250 µL of each tube's content were added to a 15 mL tube and allowed to recover by incubation at 30°C for 2 hours in a shaking incubator. From the recovered pooled libraries, 100 μL was inoculated into culture tubes containing 2 mL of either YPD for the original yeast library or SC-URA for the yeast deletion collection containing the pCYP1A2_NAT2 plasmid. Quadruplicate samples from each library were then treated with 2% MeOH, 400 μM IQ, or 800 μM IQ. Samples were incubated on a rotary incubator at 30°C for either 15 hours (for the YPD cultures) or 20 hours (for the SC-URA cultures), approximately 10 generations. The cells were then centrifuged and washed thrice with sterile water.

To amplify molecular barcodes, DNA from each treatment tube was isolated using the “smash and grab” method (Hoffman and Winston 1987) and resuspended in TE buffer (10 mM Tris–HCl, 1 mM EDTA, pH 8.0) at concentrations of 2–4 µg/µL. Polymerase chain reaction (PCR) was performed on each sample using customized oligos (Supplementary Table 2) that hybridize to the regions just upstream and downstream of the barcode “uptag” sequences; the oligos contained a multiplex index and sequencing primers for use with the Illumina Hi-Seq 2500 (Smith et al. 2009; Robinson et al. 2014). PCR protocols for barcode amplification were previously described (Robinson, et al. 2014). The ∼150 bp PCR products were resolved on a 10% polyacrylamide nondenaturing gel, extracted using the “crush and soak” method (Green and Sambrook 2019), and quantified. A pooled sample containing 50 ng of DNA from each of the 32 samples was sent to the Bauer Sequencing Core Facility at Harvard University for quality assurance and sequencing using the Illumina HiSeq 2500 platform. For experiments comparing 5-generation vs 10-generation IQ exposures, samples were sent to the University at Buffalo Genomics and Bioinformatics Core Facility for quality assurance and sequencing on the Illumina HiSeq 2000 platform.

### Barcode analysis

The sequencing data obtained in the fast all with quality (FASTQ) files was demultiplexed to separate reads from different exposures. The individual files were then subjected to a FastQC report, and then trimmed to include only the barcode region with 2–3 extra bases on either side. Barcodes were counted with an in-house program and associated with strains. Replicate treatments were grouped and compared to solvent treatment. Comparisons of treatment were performed using Bioconductor's “TCC” package in RStudio (Sun et al. 2013), processed in batches using RStudio version 1.4.1717 and R version 4.1.0 using the code in Supplementary File S1.

To determine which strains decreased in abundance after IQ exposure compared to MeOH exposure, m-values $\left(m\;=\;\log_{2}\frac{BarcodeCounts_{IQ}}{BarcodeCounts_{MeOH}}\right)$, were calculated. Negative m-values indicate that the strain was depleted after exposure and the corresponding open reading frame (ORF) or gene conferred IQ resistance. Positive m-values indicate that the strain was enriched after treatment and the corresponding ORF conferred IQ sensitivity. Q-values (modified *P*-values) less than 0.1 were deemed statistically significant. A complete list of normalized counts and processed data for all screens can be found in Supplementary File S2.

### Western blots

Expression of CYP1A2 and NAT2 in yeast cells was determined by Western blot. Cells were inoculated in YPD (Original) or SC-URA medium (Containing pCYP1A2_NAT2) and grown to log phase (A_600_ between 0.5 and 1) and then concentrated. Protein extracts were prepared as described previously by Foiani et al. (1994). Proteins were then separated on a 10% polyacrylamide gel and transferred to polyvinylidene fluoride membranes. To detect human CYP1A2, a mouse anti-CYP1A2 antibody (1:1,000, Abcam: ab22717) was used, followed by a goat antimouse secondary antibody (1:10,000, Santa Cruz Biotechnology: sc-2005). To detect human NAT2, a polyclonal mouse anti-NAT2 antibody (1:1,000, Abcam: ab88443) was used, followed by the same goat antimouse secondary antibody (1:10,000, Santa Cruz Biotechnology: sc-2005). Precision Plus Protein Western C Blotting Standards (Bio-Rad) were used for molecular weight standards.

### CYP1A2 and NAT2 enzyme activity assays

To determine whether the expressed protein was functional, the methoxyresorufin O-demethylase (MROD) activity of CYP1A2 was measured, using a method previously described (Fasullo et al. 2014). Microsomes were prepared using a modified protocol originally described by Pompon et al. (1996) and St. John et al. (2020). Microsomal protein concentrations were 15–20 mg/ml. The reaction mix consisted of adding 1 μL microsomes to 5 μM 7-methoxyresorufin in 10 mM Tris, pH 7.4 at 37°C. Reactions were started with the addition of 500 mM NADPH and fluorescence was recorded every minute for 1 hour. Fluorescence of resorufin was measured on a Tecan Infinite M200 plate reader with an excitation wavelength of 535 nm and emission wavelength of 580 nm. Microsomes derived from BY4743 containing no CYP1A2 were used for the negative control. For a positive control, rat liver S9 fractions were used.

To measure the NAT activity of NAT2, we used cytosolic extracts from yeast expressing CYP1A2 and NAT2, prepared as described by Paladino et al. (1999). Briefly, a 100 mL culture, grown to midlog phase, was pelleted and cells were washed twice before being resuspended in 2 mL ice-cold disruption buffer (100 mM Tris, pH 7.4, 100 mM DTT, 1 mM EDTA, 10% (v/v) glycerol, and 1 × yeast proteinase inhibitor cocktail IV (Thermo Scientific). Cells were lysed in the bullet blender after addition of 200 mg 0.45–0.55 mm glass beads. After 10 mins on the bullet blender, the lysate was removed to a new tube and the beads were washed twice in an equal volume of ice-cold disruption buffer. The 3 lysate fractions were pooled in a new tube. Cell debris was pelleted by centrifugation at 21,000 × *g* for 20 mins at 4°C and the supernatant was further centrifuged for 1 hour at 100,000 × *g*, before being aliquoted for assays. Extract (50 μL) was then added to a new tube containing 27.5 μL reaction buffer (225 mM Tris, pH 7.5, 4.5 mM DTT, 4.5 mM EDTA, 1 × proteinase inhibitor cocktail IV), 4.5 μL of 2 mM sulfamethazine (SMZ), and reactions were initiated with the addition of 18 μL of 2.5 mM acetyl-CoA. Negative controls included water in place of acetyl-CoA. Reactions were terminated after 30 minutes by the addition of 50 μL 25% trichloroacetic acid. To each reaction tube, 760 μL of 5% 4-dimethylamino-benzaldehyde in acetonitrile was added and mixed. Tubes were spun for 1 minute at 2,000 × *g* to remove precipitate. Aliquots of 100 μL were then added to a 96-well plate and absorbances at 450 nm were measured. The presence of acetyl-SMZ was determined by loss of signal at 450 nm.

### Growth curves

In brief, individual saturated cultures were prepared for each yeast strain. Cell density was adjusted to 0.8 × 10^7^ cells/mL for all cultures. For single mutants, strains containing pCYP1A2_NAT2 were maintained in selective medium (SC-URA), and exposed in duplicate MeOH, 400 μM IQ, or 800 μM IQ. The plate was covered with optical adhesive tape and incubated at 30°C in a TECAN Infinite M200 plate reader and absorbance at 600 nm was recorded every 10 minutes for 24 hours, as previously described (Fasullo et al. 2010; Fasullo et al. 2014; St. John et al. 2020). Absorbance was plotted against time and the areas under the curve (AUCs) were calculated for the time interval of 0 to 20 hours using freely available software (https://www.padowan.dk/download/). We calculated percent growth in the presence of toxin using the formula (AUC_IQ_/AUC_MeOH_)×100% (Toussaint et al. 2006). Statistical significance of differences between growth percentages for diploid strains and BY4743 was determined by Student's t-test, assuming constant variance between samples.

### Competitive growth assays

We measured competitive fitness of a strain using a modified protocol (North et al. 2012; De la Rosa et al. 2017). In brief, the strain was grown to a stationary phase in either YPD or SC-URA (for strains containing pCYP1A2_NAT2). In addition to the strain of interest, a wild-type strain (YB676), constitutively expressing GFP containing the pCYP1A2_NAT2 plasmid, was grown to saturation. Cells from the strain of interest and the GFP-expressing strain were combined at a set ratio and inoculated into 2 mL of culture at a concentration of 3× 10^5^ cells/mL. These cultures were then treated in duplicate with either solvent, 400 μM IQ, or 800 μM IQ and incubated for 20 hours on a rotary shaker at 30°C. Cells were then washed thrice with water and resuspended in 1 mL of water. Cells (2 × 10^4^) were then counted on an Amnis ImageStream^X^ imaging flow cytometer (*N* = 2). Cells were imaged with brightfield and a 488 nm laser at 50 mW power. The percentage of GFP-expressing cells to a total number of cells was compared for solvent-exposed and IQ-exposed cells. Data were analyzed with the IDEAS v6.2 software. In-focus cells were gated, followed by single cells, which included budded cells. GFP fluorescence intensity was quantified for the focused, single-cell population which resulted in 2 distinct peaks, 1 for fluorescent cells and 1 for nonfluorescent cells. Differences in GFP percent between IQ and methanol treatments of the strains of interest were compared to the difference in GFP percent of the IQ and methanol treatments with BY4743. The statistical significance of these differences was determined by Student's t-test, assuming constant variance between samples.

### Ames test

To ensure the mutagenicity of IQ, we performed the Ames test with the preincubation protocol (Maron and Ames 1983). In brief, cultures of *Salmonella typhimurium* TA98 and TA100 were grown overnight at 37°C to a concentration of 1–2 × 10^8^ cells/mL. The cultures were placed on ice until preincubation. An S9 master mix was made according to the protocol (33 mM KCl, 8 mM MgCl_2_, 5 mM glucose-6-phosphate, 4 mM NADP, 100 mM phosphate buffer pH 7.4), and to it was added rat liver S9 (4%) or yeast-derived microsomes, and 495 μL of this master mix were added to culture tubes, in triplicate, along with 100 μL of the chilled bacteria. IQ was added at either 4 or 40 µM concentration before incubating the tubes in a heat block at 37°C for 20 minutes. After incubation, 2 mL of molten 0.6% top agar, heated to 48°C, was added and the mix was pipetted onto minimal glucose agar plates. Plates were cooled for 5 minutes, then inverted and incubated for 48 hours at 37°C before His^+^ colonies were counted.

### Trypan blue staining

To measure cell viability after IQ exposure, we performed a trypan blue exclusion assay. Selected strains expressing CYP1A2 were inoculated in SC-URA until cultures reached an A_600_ of 0.1–0.5, and then exposed to either 800 μM IQ or 2% solvent alone. After incubating for 3 hours, cells were washed twice in sterile phosphate-buffered saline (PBS) and stained with trypan blue at a final concentration of 2 mg/ml (Liesche et al. 2015). Both the total number and blue-stained (dead) cells were counted on a light microscope using a hemocytometer. At least 200 cells were counted for each experiment, and percentages of live and dead cells were calculated, *N =* 3. The differences between percent dead cells after IQ treatment and methanol treatment were calculated. To determine statistical significance, the differences in percent dead for each strain of interest were compared to the difference in percent dead in wild type (WT, YB679, *N* = 10) using Student's 1-tailed *t*-test.

## Results

### Expression of CYP1A2 and NAT2 enhances IQ toxicity in yeast rad^+^ and *rad4 rad51* mutants

**Fig. 2.**
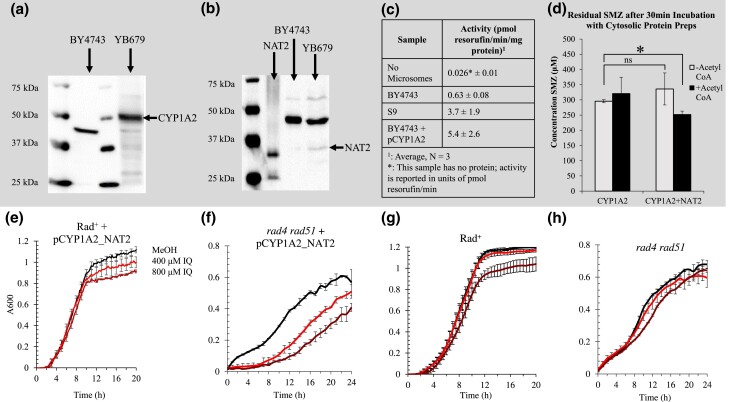
IQ sensitivity and expression of CYP1A2 and NAT2 in rad^+^ and DNA repair deficient strains. Panel A is a Western blot that detects the human CYP1A2 protein present in yeast protein extracts. The black arrow indicates the position of CYP1A2, which is ∼58 kDa. The first and third lanes from the left contain the molecular weight standards. The second lane contains the protein extract from wild type (BY4743) and the last lane contains the extract from wild-type strain containing pCYP1A2_NAT2 (YB679). Panel B is a Western blot that detects the human NAT2 protein in yeast protein extracts. The red arrow indicates the position of the NAT2 protein, which is ∼37 kDa. The first lane from the left contains the molecular weight standards. The second contains a purified NAT2 protein. The third and fourth lanes, respectively, contain the wild-type (BY4743) and the wild type expressing pCYP1A2_NAT2 (YB679). Panel C shows the 7-MROD activity of CYP1A2 ±1 standard deviation, *N* = 3. Panel D shows the acetyltransferase activity of Nat2. Acetyl-CoA is a cofactor, without which the reaction does not occur. Error bars represent 1 standard deviation, *N* = 3. Panels E-H are growth curves of Rad^+^ or *rad4  rad51* yeast strains exposed to 2% MeOH (black), 400 µM IQ (Light red), or 800 µM IQ (Dark red), plotted as absorbance (A_600_) against time (Hours). Error bars represent 1 standard deviation at 1-hour intervals. Panel E is a growth curve of the Rad^+^ diploid strain containing pCYP1A2_NAT2 (YB679), *N* = 8. Percent growth with 400 µM IQ and 800 µM IQ were 94 and 83%, respectively. Panel F is a growth curve of the *rad4  rad51* haploid yeast strain containing pCYP1A2_NAT2 (YB400), *N* = 3. Percent growth with 400 µM IQ and 800 µM IQ were 60 and 41%, respectively. Panel G is a growth curve of the Rad^+^ diploid strain (BY4743), *N* = 8. Percent growth with 400 µM IQ and 800 µM IQ were 99 and 93%, respectively. Panel H is a growth curve the *rad4  rad51* (YB226) strains, *N* = 3. Percent growth with 400 µM IQ and 800 µM IQ were 94 and 87%, respectively.

To confirm that the expression of CYP1A2 and NAT2 bioactivate IQ, the expression plasmid pCYP1A2_NAT2 was introduced in wild type yeast (BY4743) and a *rad4  rad51* strain by selecting Ura + transformants. The *rad4  rad51* strain is defective in both nucleotide excision and recombinational repair and is sensitive to DNA damaging agents that form bulky DNA adducts as well as DNA strand breaks. Expression of CYP1A2 and NAT2 proteins was then confirmed by Western blots (Fig. 2a and 2b, respectively). We performed MROD assays to quantify CYP1A2 activity (Fig. 2c, Fasullo et al. 2014) and NAT assays to detect NAT2 activity (Fig. 2d). Yeast extracts from these assays also activated IQ in the Ames assay; compared to extracts from the strain expressing no human genes, the mutagenicity of IQ was highest (*P* < 0.05, Supplementary Table 3) when IQ was pre-incubated using microsomal and cytosolic extracts obtained from strains expressing CYP1A2 and NAT2, but still detectable using extract from strains expressing CYP1A2 alone (Supplementary Table 3). These data indicate that CYP1A2 and NAT2 were expressed in yeast and were sufficient to increase the mutagenicity of IQ.

By calculating AUCs, we determined IQ concentrations that reduced growth in both the wild-type Rad + diploid (BY4743) and the *rad4  rad51* haploid mutant, which either did (“humanized”) or did not (original) express CYP1A2 and NAT2. Exposure to 400 μM IQ and 800 μM IQ, reduced growth of the “humanized” Rad + diploid by 6 and 17%, respectively (Fig. 2e), and in the “humanized” *rad4  rad51* haploid by 40 and 59%, respectively (Fig. 2f). On the other hand, exposure to 400 μM IQ and 800 μM IQ, reduced the growth of the original wild type by 1 and 7%, respectively (Fig. 2g), and in the original *rad4  rad51* haploid by 6 and 13% (Fig. 2h). In comparison to the Rad + diploid (BY4743), the higher level of IQ toxicity in the “humanized” Rad + diploid is significant only for the 800 µM IQ exposure (*N* = 8, *P* = 0.035). In comparison to *rad4  rad51*, the higher level of IQ toxicity in the “humanized” *rad4  rad51* is significantly different both at 400 µM IQ (*N* = 3, *P* = 0.019) and 800 µM IQ (*P* = 0.007) exposure. These data indicate the “humanized” strains exhibit a higher level of IQ genotoxicity.

### Identification of resistance genes by barcode analysis

**Fig. 3.**
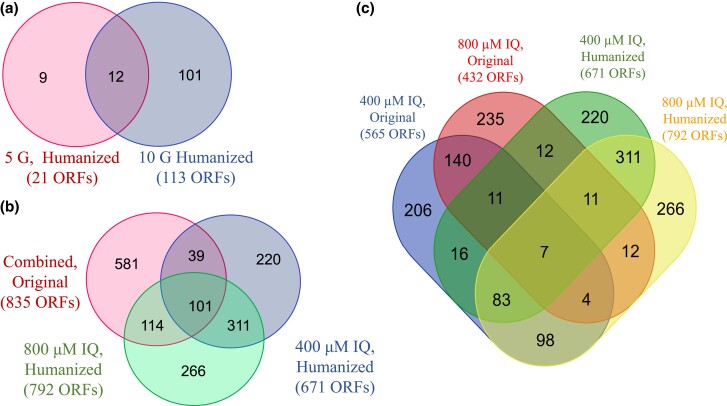
Venn diagrams of IQ resistance genes identified in screens that vary either exposure time or IQ concentrations. All ORFs were identified from the yeast diploid deletion library expressing CYP1A2 and NAT2. ORFs include genes of known function, genes of unknown function, and uncharacterized open Reading frames, which confer IQ resistance. Panel A is the overlap of the ORFs identified after 800 µM IQ exposure for 5 and 10 generations. Twenty-one ORFs were identified after 5 generations and 113 ORFs were identified after 10 generations. Panel B is the overlap of the IQ resistance genes identified after 400 µM and 800 µM IQ exposures for 10 generations. Individually, 671 ORFs were identified after 400 µM IQ exposure and 792 ORFs were identified after 800 µM IQ exposures. The original library treated with either IQ concentration identified 835 unique ORFs across both 400 µM and 800 µM IQ treatments. Panel C expands the combined original library from B into ORFs from the individual 400 µM (565 ORFs) and 800 µM IQ exposures (432 ORFs), displaying the degree of overlap.

We identified genes that confer resistance to either IQ per se, or activated IQ in 6 independent screens (Fig. 3). We exposed the original pooled diploid collection to 400 μM and 800 μM IQ for 10 generations (10G). We also exposed the pooled “humanized” yeast diploid collection to 400 μM and 800 μM IQ for 10G. In addition, we exposed the “humanized” collection to 800 μM IQ exposure for 5 generations (5G) and 10G. The control for all screens was exposed to 2% methanol with 0.1% acetic acid (MeOH) and 4 biological replicates were performed for each exposure and 2 technical replicates were sequenced. Approximately 98% (4513**/**4607) of strains from the original library and 89% (4343/4900) of strains from the “humanized” library were detected in the pooled cultures. Genes conferring IQ resistance were identified by differential abundance calculations of barcode counts where m-values <0 and q-values <0.1.

Across 4 screens of the “humanized” library, 1159 ORFs from the “humanized” library were identified that conferred IQ resistance at any condition (see Supplementary Table 4), of which, 424 ORFs were identified in at least 2 independent screens. In the time-dependent study, 12/21 (57%) of the ORFs identified at 5G were present in the 10G (113 ORFs) dataset (Fig. 3, Panel A). In the concentration-dependent study, 412/671 (62%) ORFs from the 400 µM screen were also present in the 800 µM dataset (792 ORFs) (Fig. 3, Panels b and c). These data thus show considerable overlap between screens of the “humanized” library.

A random selection of the concentration-dependent screen was used to estimate false positives and false negatives. To estimate the number of false positives among the overlapping set of 412 ORFs conferring IQ resistance in the concentration-dependent study, ORFs were alphabetized and 40 were randomly selected. Of these 40, 39 (98%) strains exhibited sensitivity to IQ in the trypan blue assay (Supplementary Table 5). Strains not identified as significant (2,049 strains) were also alphabetized and 10 strains were randomly selected; of these, 9 were not significantly IQ sensitive (Supplementary Table 5). We thus estimate that our false positive percentage was less than 5%, while our failure to detect IQ-sensitive strains was approximately 10%.

### ORFs conferring resistance in the “humanized” library

**Fig. 4.**
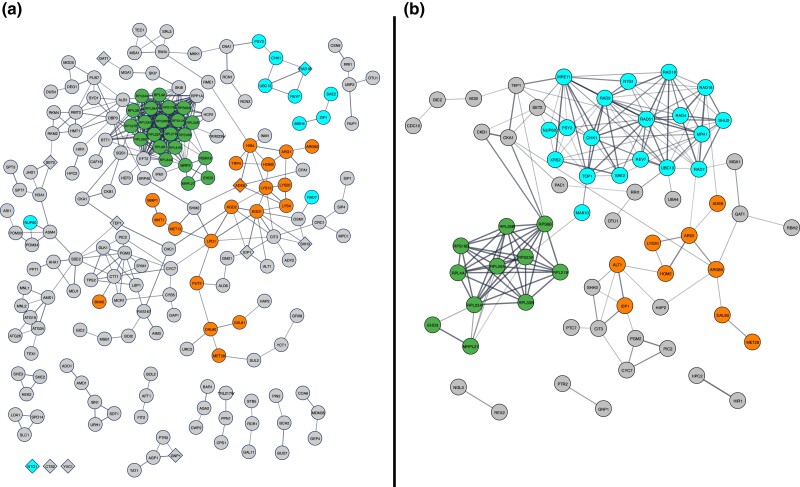
Protein interactome identified from screens of the diploid deletion library containing pCYP1A2_NAT2. The interactome was curated using String V11 (https://string-db.org, Szklarczyk et al. 2019). Nodes represent proteins, while edges represent interactions between 2 proteins. More evidence of interaction between proteins is denoted by thicker lines. Colored nodes indicate proteins involved in translation (green), DNA damage tolerance (blue), and amino acid metabolism (orange). Genes with human orthologs implicated in colon cancer are shown as diamond-shaped nodes. Disconnected nodes have been removed to improve readability. Panel A represents the functional interactome of proteins encoded by the 424 ORFs that were identified in both screens of the “humanized” library. Disconnected nodes in the bottom left were included because they have human orthologs implicated in colorectal cancer (CRC). Protein interactions with “high” confidence (0.7) of interaction are displayed. Panel B represents the functional interactome of each protein encoded by the 93 ORFs from the “humanized” yeast deletion library, which have been individually validated. Protein interactions with a “medium” confidence (0.4) of interaction are displayed.

Based on our estimate that over 95% of duplicated ORFs confer IQ resistance in the “humanized” library, we identified key interactions that mediate IQ resistance from our list of 424 ORFs (Fig. 4a), using ELIXIR's Core Data Resource STRING (https://string-db.org/, Szklarczyk et al. 2019 ). A network of proteins from ORFs conferring IQ resistance in at least 1 screen is presented in Supplementary Fig. 1. In both sets, the largest and most heavily connected cluster was ribosomal proteins (Fig. 4a, Supplementary Fig. 1). Additional clusters in both sets are encoded by DNA damage tolerance genes, such as *REV7*, *UBC13*, and *RAD18*, and nitrogen metabolism genes, such *ALT1*, *ADE6*, and *LYS20*. These data thus illustrate several key gene interaction networks involved in protein metabolism and DNA damage repair.

Genes selected for validation included those from the major interactome clusters, genes that were missing from the library but whose presence was implied based on related significant genes, as well as genes with ambiguity in whether they conferred resistance or sensitivity based on the outcomes of multiple screens. We validated IQ resistance genes using growth curves, competitive growth assays, and trypan blue staining; while growth curves and competitive growth assays measure toxin resistance after chronic exposures, trypan blue was used to determine cell viability after an acute 3 h IQ exposure. The interactome of proteins from all validated ORFs (Fig. 4b) contains a larger cluster of DNA repair genes than the interactome of proteins from all ORFs appearing at least twice (Fig. 4b).

In total, we tested 51 selected genes by both competitive growth and trypan blue assays, of which, 18 were also tested by growth curves (Table 1). Twenty-one genes were validated by only 1 method, 30 (59%) were validated by at least 2 methods, and 7 were validated by all 3. We previously observed that after 10 generations, 10–40% of total cells, depending on the strain, still lose the pCYP1A2_NAT2 plasmid, even in selective media, rendering it difficult to accurately determine toxin sensitivity by trypan blue viability or growth curves (St. John et al. 2020). On the other hand, CYP1A2 expression increases the number of nonfluorescent cells in the “wild-type” strain containing the GFP construct ([6 ­ ± 4] %, *N* = 28) thus undercounting the true number of “wild-type” cells after competitive growth. This may account for why over half the validated genes (8 out of 15) that have m-values greater than −0.7 did not validate by competitive growth assays. Nonetheless, despite these limitations, most genes are validated by multiple independent methods for monitoring toxin sensitivity.

**Table 1.** Validation of 51 genes conferring IQ resistance.

			Competitive growth		Trypan blue	
Strain*^a^*	M-Value (800 µM)	q-Value*^b^* (800 µM)	% GFP (difference)*^c^*	*P*-value*^d^*	% positive (difference)*^e^*	*P*-value*^f^*
BY4743	-	-	12% (0.8%)	-	4.2% (0.9%)	-
*REV7*	−3.66	3.12E-03	9.8% (1.3%)	1.95E-01	**13.0% (4.6%)**	6.23E-04
*UBC13*	−3.62	1.55E-03	**17.0% (3.8%)**	1.49E-02	**21.7% (6.7%)**	2.18E-05
*RPL21B*	−3.61	1.37E-04	**7.7% (2.6%)**	4.28E-03	**15.8% (4.5%)**	1.27E-04
***SAE2***	−3.34	7.64E-04	**8.2% (1.4%)**	3.86E-02	**10.2% (2.7%)**	2.73E-02
*CDC10*	−3.33	1.06E-03	**73.7% (7.8%)**	1.14E-06	**51.4% (24.7%)**	5.28E-12
*HOM2*	−3.06	1.43E-03	14.0% (−1.2%)	3.36E-01	**30.7% (7.5%)**	5.49E-03
*CHK1*	−3.03	3.96E-02	5.1% (0.8%)	4.66E-01	**31.0% (9.6%)**	1.03E-06
*IDP1*	−2.99	2.63E-03	**18.7% (3.0%)**	2.24E-03	**34.5% (7.7%)**	1.30E-05
*RAD7*	−2.73	5.68E-04	11.3% (1.5%)	1.44E-01	**11.1% (3.5%)**	9.54E-03
*CKB1*	−2.04	1.46E-02	**6.5% (2.5%)**	2.05E-02	6.0% (1.4%)	3.06E-01
*PSY2*	−1.76	6.59E-02	6.9% (1.2%)	2.50E-01	**51.1% (19.8%)**	1.21E-10
*CKA1*	−1.51	7.63E-02	**10.4% (2.9%)**	3.70E-03	**24.7% (9.8%)**	5.19E-07
*HIR1*	−1.42	4.49E-02	**10.4% (1.8%)**	3.72E-02	**12.4% (3.4%)**	6.92E-03
*MET28*	−1.42	4.39E-02	**10.4% (2.8%)**	6.66E-03	**12.9% (6.7%)**	1.33E-05
*SWT21*	−1.40	1.00E-01	**12.6% (0.1%)**	7.54E-02	**25.4% (5.3%)**	2.22E-04
*NGL3*	−1.38	*1.42E-01*	**7.8% (0.1%)**	1.92E-02	**15.4% (3.8%)**	1.57E-03
***RPL33B***	−1.26	6.31E-05	**19% (7.6%)**	5.99E-07	**9.3% (2.2%)**	4.59E-02
***ADE6***	−1.25	3.81E-03	**7.7% (2.6%)**	4.63E-05	**12.1% (7.6%)**	6.98E-06
*PTR2*	−1.23	3.31E-02	7.6% (1.4%)	1.76E-01	**8.5% (3.0%)**	5.40E-03
*RPL23A*	−1.19	2.62E-03	**19.7% (4.4%)**	3.59E-05	**27.6% (8.9%)**	9.16E-06
*RPS16B*	−1.13	4.09E-03	**1.7% (0.1%)**	4.84E-02	**8.3% (2.6%)**	1.46E-02
*GAT1*	−1.08	1.69E-03	**21.1% (5.3%)**	1.86E-04	**5.8% (1.4%)**	2.73E-02
*HPC2*	−1.07	4.02E-02	**10.2% (2.9%)**	9.82E-04	**18.4% (8.1%)**	3.30E-06
*SHH3^g^*	−1.04	5.59E-03	**11.0% (1.7%)**	2.60E-03	**22.1% (11.3%)**	4.44E-05
***RAD18^g^***	−1.02	3.09E-02	**26.8% (8.7%)**	2.19E-05	**14.3% (11.5%)**	4.74E-08
*RPL4A*	−1.02	1.60E-02	5.4% (0.9%)	4.63E-01	**9.2% (2.8%)**	1.60E-02
*CTS2*	−1.01	3.22E-02	**35.7% (21.0%)**	1.50E-11	**4.6% (3.3%)**	1.34E-02
*NTG1^g^*	−1.00	2.45E-02	**13.5% (2.6%)**	1.07E-02	**12.6% (4.8%)**	2.01E-03
*GNP1^g^*	−0.923	2.54E-02	**37.4% (11.3%)**	1.11E-05	**16.1% (3.5%)**	5.18E-03
*EHD3*	−0.882	5.81E-04	**8.6% (2.8%)**	4.53E-03	**17.0% (4.0%)**	3.74E-05
*ARG1*	−0.873	3.15E-05	**23% (6.2%)**	2.47E-06	**40.8% (7.3%)**	1.23E-05
*CYC7*	−0.848	5.05E-03	**8.4% (2.4%)**	9.28E-03	**26.4% (8.6%)**	1.86E-07
*RPL26B*	−0.735	1.29E-04	**21.9% (5.1%)**	1.73E-03	**8.1% (4.9%)**	2.66E-04
***RPL26A***	−0.660	9.49E-05	2.8% (−0.5%)	1.75E-02	**16.9% (5.4%)**	1.44E-03
*CWP2*	−0.646	3.49E-02	4.9% (0.0%)	9.98E-02	**31.5% (11.8%)**	4.70E-07
*RPS6B*	−0.644	*1.41E-01*	**5.4% (1.5%)**	1.36E-01	**6.6% (5.4%)**	4.94E-06
*RAD9*	−0.618	*1.28E-01*	**24.0% (4.1%)**	5.77E-05	**16.3% (6.9%)**	1.32E-04
*DAL80*	−0.559	2.80E-04	10.2% (1.8%)	7.12E-02	**15.1% (6.2%)**	3.91E-05
*RAD51*	−0.558	*7.85E-01*	**66.5% (26.4%)**	9.56E-12	**21.0% (13.0%)**	1.25E-04
*ARG80*	−0.532	9.18E-03	**7.9% (1.2%)**	2.12E-01	**25.8% (10.7%)**	8.98E-08
***SET2***	−0.516	2.54E-02	**34.3% (8.7%)**	4.04E-05	**11.9% (6.5%)**	1.78E-05
*EMP46*	−0.504	9.33E-03	15.8% (−0.2%)	3.11E-01	**23.0% (6.0%)**	6.69E-05
*TEP1*	−0.503	3.78E-02	**21.1% (3.3%)**	3.50E-02	**9.5% (4.8%)**	3.95E-04
***MPH1***	−0.489	*1.38E-01*	**11.6% (2.7%)**	2.50E-03	7.8% (2.8%)	7.06E-02
***ALT1***	−0.480	2.01E-03	**17% (11.4%)**	1.49E-09	**8.7% (4.1%)**	1.44E-03
*TOP1*	−0.458	3.36E-03	6.1% (0.9%)	1.89E-01	**13.7% (4.9%)**	6.71E-05
*CIT3*	−0.367	3.68E-02	**11.6% (2.2%)**	1.83E-02	**39.2% (12.7%)**	9.37E-08
*PTC7*	−0.292	*1.09E-01*	8.0% (1.0%)	3.35E-01	**11.5% (2.8%)**	2.63E-02
*MMP1*	−0.276	3.73E-02	23.2% (1.7%)	8.02E-02	**14.2% (4.8%)**	1.06E-02
*LYS20*	−0.256	6.20E-02	**12.9% (7.0%)**	1.10E-06	**9.7% (2.2%)**	8.22E-02
*RPS23A*	−0.166	4.46E-02	5.1% (−0.4%)	3.55E-03	**7.2% (2.9%)**	8.35E-03

^*a*^Bolded genes were showed statistically significant differences in AUCs in growth curves.

^*b*^Italicized values did not meet the threshold of q < 0.1 in 800 µM study.

^*c*^Difference = percent of GFP-expressing cells when treated with IQ minus percent GFP-expressing cells when treated with MeOH; Bolded values indicate agreement that a gene confers resistance with *P* < 0.05.

^*d*^Significance determined by comparing difference of tested strain with difference in the wild-type.

^*e*^Difference = percent dead cells when treated with IQ minus percent dead cells when treated with MeOH; Bolded values indicate agreement that a gene confers resistance with *P* < 0.05.

^*f*^Significance determined by comparing differential death of tested strains with the wild-type.

^*g*^m-values and q-values from 10G screen, from which these genes were identified.

**Fig. 5.**
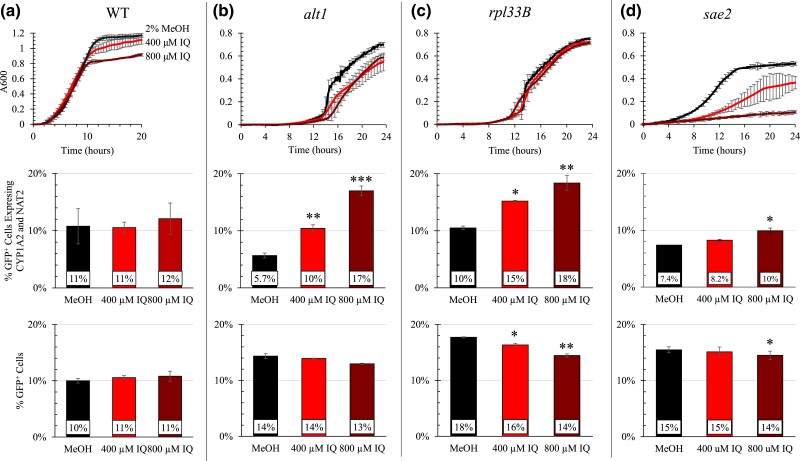
Growth curves and competition assays for selected IQ resistance genes identified in high-throughput screens. Panels A, B, C, and D show the wild-type strain, *alt1*, *rpl33B*, and *sae2*, respectively. The latter 3 represent major gene clusters highlighted in Fig. 4, including nitrogen metabolism (*ALT1*), ribosome protein (*RPL33B*) and DNA repair (*SAE2*). The top row contains growth curves for selected strains containing pCYP1A2_NAT2. A_600_ is plotted against time (Hours) with the background absorbance was subtracted, *N* ≥ 3. Error bars represent 1 standard deviation for 1-hour measurements. The middle row shows competitive growth data for the indicated strains containing pCYP1A2_NAT2. The y-axis shows the percent of GFP-containing wild type expressing CYP1A2 and NAT2 (YB676). Competitive cultures were inoculated into SC-Ura at approximately 10% YB676 and 90% the indicated strain. 2 × 10^4^ cells were acquired per sample. Error bars represent 1 standard deviation. For wild type (YB679), *N* ≥ 5; for all others, *N* = 2. Significance was determined by Dunnett's test and asterisks indicate *P* < 0.05 (*), *P* < 0.01 (**) and *P* < 0.001(***). The bottom row shows competitive growth data for the indicated strains not expressing CYP1A2 or NAT2. The y-axis shows the % of GFP-containing WT (YB675). Competitive cultures were inoculated into SC at approximately 10% YB675 and 90% the indicated strain and grown for 20 hours. 2 10^4^ cells were acquired per sample. For wild type (BY4743), *N* = 12; for all others, *N* = 2. Error bars represent 1 standard deviation. Significance was determined by Dunnett's test and asterisks indicate *P* < 0.05 (*), *P* < 0.01 (**) and *P* < 0.001(***).

As an example, mutants representative of the ribosome (*rpl33b*), nitrogen metabolism (*alt1*), and DNA repair (*sae2*) clusters, exhibited particularly high IQ sensitivity either by growth curves or competitive growth assays, as well as validating with trypan blue (Fig. 5). *ALT1* functions in utilizing alanine as a nitrogen source, and *alt1* mutants are sensitive to oxidative stress. *RPL33B* encodes the ribosomal 60S genes and is essential when its paralog *RPL33A* is defective. *SAE2* is an endonuclease involved in several DNA repair and maintenance functions. Only significant ORFs conferring resistance in at least 1 screen and which were validated by at least 1 method are included in further analysis (82 ORFs).

We identified protein complexes using the Princeton gene ontology (GO) Term finder (https://go.princeton.edu/cgi-bin/LAGO, Boyle et al. 2004). Protein complexes in global cell regulation and housekeeping were identified among the validated ORFs using the Princeton GO Term finder (*P* < 0.05), and redundancy was reduced using Revigo (Supek et al. 2011). The resulting 5 components GO groups included the nonmembrane bound organelle, ribosome, ribosomal subunit, the protein kinase CK2 complex, and the HIR complex (Table 2). The CK2 kinase complex regulates cell cycle progression, while the HIR complex, also referred to as the replication-independent HIR complex, regulates chromatin structure and affects gene expression. These complexes underscore the importance of regulating cell cycle progression and protein translation and gene expression in IQ resistance. For all GO Terms from the complete set of overlapping ORFs, see Supplementary Table 6.

**Table 2.** Component GO terms for validated IQ resistance genes.

Term ID	Description	*P*-value*^a^*	Annotations*^b^*	Annotated genes*^c^*
GO:0044391	Ribosomal subunit	2.67E-05	12 out of 250	*EHD3, MRP35, MRPL27, RPL21B, RPL23A, RPL26A, RPL26B, RPL33B, RPL4A, RPS16B, RPS23A, RPS6B*
GO:0005840	Ribosome	7.04E-05	12 out of 276	*EHD3, MRP35, MRPL27, RPL21B, RPL23A, RPL26A, RPL26B, RPL33B, RPL4A, RPS16B, RPS23A, **RPS6B***
GO:0000417	HIR complex	1.30E-03	2 out of 4	*HIR1, HPC2*
GO:0005956	Protein kinase CK2 complex	1.93E-03	2 out of 4	*CKA1, CKB1*
GO:0043228	Nonmembrane-bounded organelle	4.83E-03	29 out of 1,657	*CDC10, CHK1, CKA1, CKB1, EHD3, GIC2, HIR1, HPC2, IDP1, MGA1, **MPH1**, MRP35, MRPL27, NBA1, NKP1, PAC1, PSY2, **RAD18**, PL21B, RPL23A, RPL26A, RPL26B, RPL33B, RPL4A, RPS16B, RPS23A, **RPS6B**, SET2, **TOP1***

^*a*^P-value with applied cutoff of 0.01.

^*b*^Genes from the 82 validated ORFs out of the total number of yeast ORFs belonging to each GO group, based on reference list of 7166 ORFs.

^*c*^Genes were significant in at least 2 screens and were independently validated as conferring IQ resistance.

Bolded genes were significant in at least 1 screen, and were independently validated as conferring IQ resistance.

In addition to the protein complexes, we identified process and function GO groups represented by the 82 ORFs (*P* < 0.01) using the Princeton LAGO tool and reduced redundancy using Revigo, resulting in 4 “functions” and 16 “processes” (Table 3). Key constituents that were expected included the structural constituent of the ribosome, DNA repair, DNA damage response, and nitrogen cycle metabolic process GO terms. Other terms included alpha-amino acid metabolic process, regulation of nucleobase-containing compound metabolic process, DNA binding, regulation of ergosterol biosynthetic process, and regulation of transcription by RNA polymerase III. These process terms further emphasize the role of nitrogen metabolism in conferring IQ resistance, as well as the role of nucleic acid binding.

**Table 3.** Function and process GO terms for validated IQ resistance genes.

Term ID	Description	*P*-value*^a^*	Annotations*^b^*	Annotated genes*^c^*
Function				
GO:0003735	Structural constituent of ribosome	1.44E-05	12 out of 235	*EHD3, MRP35, MRPL27, RPL21B, RPL23A, RPL26A, RPL26B, RPL33B, RPL4A, RPS16B, RPS23A, **RPS6B***
GO:0005198	Structural molecule activity	9.85E-05	14 out of 379	*CDC10, CWP2, EHD3, MRP35, MRPL27, RPL21B, RPL23A, RPL26A, RPL26B, RPL33B, RPL4A, RPS16B, RPS23A, **RPS6B***
GO:0046912	Acyltransferase activity, acyl groups converted into alkyl on transfer	5.62E-03	2 out of 10	*CIT3, LYS20*
GO:0003677	DNA binding	8.87E-03	14 out of 667	*ARG80, DAL80, GAT1, HIR1, HPC2, MET28, MGA1, **MPH1**, NTG1, **RAD18**, RAD7, SAE2, **TOP1**, WAR1*
Process				
GO:1901605	Alpha-amino acid metabolic process	1.97E-04	9 out of 180	*ADE6, ALT1, ARG1, ARG80, EHD3, HOM2, IDP1, LYS20, MET28*
GO:0006974	DNA damage response	2.74E-04	13 out of 372	*CHK1, CKA1, CKB1, LYS20, **MPH1**, **NTG1**, PSY2, **RAD18**, RAD7, REV7, SAE2, SET2, UBC13*
GO:0006359	Regulation of transcription by RNA polymerase III	3.80E-04	3 out of 13	*CKA1, CKB1, HIR1*
GO:0032502	Developmental process	4.45E-04	10 out of 245	*ATG26, CDC10, CKA1, CTS2, GIC2, **MPH1**, PMD1, SAE2, SET2, TEP1*
GO:0032443	Regulation of ergosterol biosynthetic process	1.26E-03	2 out of 5	*DAP1, RRI1*
GO:0022413	Reproductive process in single-celled organism	1.57E-03	7 out of 150	*ATG26, CKA1, **MPH1**, PMD1, SAE2, SET2, TEP1*
GO:0071941	Nitrogen cycle metabolic process	2.62E-03	2 out of 7	*ARG1, DAL80*
GO:0006281	DNA repair	3.67E-03	10 out of 324	*LYS20, **MPH1**, **NTG1**, PSY2, **RAD18**, RAD7, REV7, SAE2, SET2, UBC13*
GO:0062012	Regulation of small molecule metabolic process	4.50E-03	4 out of 59	*ARG80, DAP1, MET28, RRI1*
GO:0022616	DNA strand elongation	4.69E-03	3 out of 30	***MPH1*** *, SAE2, TOP1*
GO:0006301	Postreplication repair	5.15E-03	3 out of 31	***RAD18*** *, REV7, UBC13*
GO:0006099	Tricarboxylic acid cycle	6.15E-03	3 out of 33	*CIT3, IDP1, **SHH3***
GO:0090293	Nitrogen catabolite regulation of transcription	9.30E-03	2 out of 13	*DAL80, GAT1*
GO:0019219	Regulation of nucleobase-containing compound metabolic process	9.81E-03	16 out of 749	*ARG80, CKA1, CKB1, DAL80, GAT1, HIR1, HPC2, MET28, MGA1, **MPH1**, **NTG1**, OTU1, PSY2, SET2, **TOP1**, WAR1*

^*a*^P-value with applied cutoff of 0.01.

^*b*^Genes from the 82 validated ORFs out of the total number of yeast ORFs belonging to each GO group, based on reference list of 7166 ORFs.

^*c*^Genes were significant in at least 2 screens and were independently validated as conferring IQ resistance.

Bolded genes were significant in at least 1 screen and were independently validated as conferring IQ resistance.

Fourteen IQ-validated resistance genes that function in either DNA replication checkpoints, DNA repair, or DNA damage-associated gene expression (Cherry et al. 2012) are listed in Table 4. While NER genes were not prominent, we identified the dual function base excision repair gene, *NTG1*. Several genes are directly associated with DNA damage tolerance, such as *UBC13*, *REV7*, and *RAD18*, while others are involved in cell cycle progression in the presence of DNA damage, including *PSY2*, *CKA1,* and *CKB1*. *ADE6,* which functions in de novo purine synthesis, suppresses chromosome loss, as revealed in a *MAT***a**-like faker screen (ALF, Yuen et al. 2007). Recombinational repair genes include *SAE2*. Although *RAD52*, *MRE11*, and *XRS2* were missing in all the screens of the “humanized” library, the *rad52, mre11, xrs2* strains were subsequently observed to be IQ sensitive by trypan blue assay. *MPH1* functions in blocking recombination and facilitating replication (Prakash et al. 2009). *CHK1* is a checkpoint signaling genes downstream of *RAD9* (For review, see Chen and Sanchez 2004); while *RAD9* was not initially identified in the screen, the *rad9* mutant is IQ sensitive by both trypan blue and competitive growth assays. These genes emphasize the role of postreplication repair in conferring IQ resistance.

**Table 4.** Validated checkpoint, DNA damage repair, and DNA damage-inducible genes.

ORF	Gene name*^a^*	Gene Ontology (Term ID)
YBR274W	*CHK1*	DNA damage checkpoint signaling (GO:0000077)
YIL035C	CKA1*	DNA damage response (GO:0006974)
YGL019W	CKB1*	DNA damage response (GO:0006974)
YDL182W	*LYS20*	DNA repair (GO0006281)
YIR002C	*MPH1*	DNA recombination, DNA repair (GO0006281), DNA-templated DNA replication maintenance of fidelity (GO:0045005), double-strand break repair via homologous recombination (GO:0000724), interstrand cross-link repair (GO:0036297), recombinational repair (GO:0000725)
YAL015C	*NTG1*	Base-excision DNA repair (GO0006281)
YNL201C	*PSY2*	DNA damage checkpoint signaling (GO:0000077), DNA repair (GO0006281), double-strand break repair via nonhomologous end joining (GO:0006303), signal transduction in response to DNA damage (GO:0042770)
YCR066W	*RAD18*	DNA synthesis involved in DNA repair (GO0006281), error-free postreplication DNA repair (GO0006281)
YJR052W	*RAD7*	DNA repair (GO0006281), NER DNA damage recognition (GO0000715)
YDR217C	*RAD9*	DNA damage checkpoint signaling (GO:0000077), DNA repair (GO0006281), DNA strand resection involved in replication fork processing (GO:0110025), double-strand break repair (GO:0006302), signal transduction in response to DNA damage (GO:0042770)
YIL139C	*REV7*	DNA repair (GO0006281), DNA synthesis involved in DNA repair (GO0006281), postreplication repair (GO:0006301)
YGL175C	*SAE2*	DNA double-strand break processing involved in repair via synthesis-dependent strand annealing (GO:0010791), DNA recombination (GO:0006310), DNA repair (GO0006281), double-strand break repair (GO:0006302), recombinational repair (GO:0000725)
YJL168C	*SET2*	DNA recombination (GO:0006310), DNA repair (GO0006281), transcription coupled nucleotide-excision repair (GO:0006283)
YDR092W	*UBC13*	DNA repair (GO0006281), postreplication repair (GO:0006301)

^*a*^Asterisk indicates genes not directly involved in DNA repair, but whose expression is induced in response to DNA damage.

### ORFs conferring resistance in the original yeast library

To distinguish between ORFs that confer resistance to activated IQ or IQ per se, we screened the original yeast deletion library, lacking human CYP1A2 and NAT2, for IQ resistance. We identified 835 ORFs conferring resistance to either 400 μM or 800 μM IQ, of which 143 ORFs were identified in both screens (Fig. 3, Supplementary Table 7). In the interactome of overlapping ORFs in the original library, the prominent clusters are related to asparagine-linked glycosylation, and the Rpd3L histone deacetylase (Supplementary Fig. 2a). The over-represented GO terms in the screens of the original library were negative regulation of nucleobase-containing compound metabolic process (GO:0045934) and cellular protein modification process (GO:0006464). The protein interactome of all 835 ORFs further expands the clusters of protein modification process and glycosylation, and clearly denotes ribosomal proteins, vacuolar ATPase, and DNA damage repair clusters (Supplementary Fig. 2b). These ORFs indicate protein synthesis and modification are prominent in conferring IQ resistance.

There were 254 ORFs in at least 1 screen of the “humanized” library which overlapped with at least 1 screen of the original library (Supplementary Tables 4 and 7). Of these, notable GO terms were negative regulation of DNA recombination, nucleotide-excision repair, DNA damage recognition, lipid binding, and vacuole (Supplementary Table 8). The m-values of 211 ORFs (83%) were lower from the “humanized” library than the original library (Supplementary File S2). The percentage was slightly greater (88%, 14/16) when comparing DNA damage repair genes. These data suggest there is a subset of genes required for conferring resistance to both IQ per se and “metabolically activated” IQ.

When comparing the interactomes of the unique ORFs identified in the “humanized” (Supplementary Fig. 1) and original (Supplementary Fig. 2b) libraries, the ribosome cluster is prominent in both; 15 genes were identified in the original library, while 38 were identified “humanized” library. Four ribosomal genes, *RPL22A*, *RPL26A, RPL37B,* and *RPL6B*, were identified in profiling both the “humanized” yeast library and the original library. These data suggest that activated IQ may target different subunits of the ribosome than IQ exposure per se.

### ORFs conferring sensitivity to activated IQ

We also identified ORFs conferring sensitivity to IQ (m > 1, q < 0.1, Supplementary Tables 4 and 7). While 54 were identified in duplicate screens of the original library, 502 were identified in duplicate screens of the “humanized” library. Genes conferring IQ sensitivity included those that function in nitrogen compounds and organic substance transport (19%); we speculate that transporting IQ into cellular compartments may enhance sensitivity. Genes that confer IQ sensitivity may also be required for or enhancing expression of CYP1A2 or NAT2, such as those encoding cytochrome B reductase (*CBR1*) and cytochrome C oxidase (*COX14*).

**Fig. 6.**
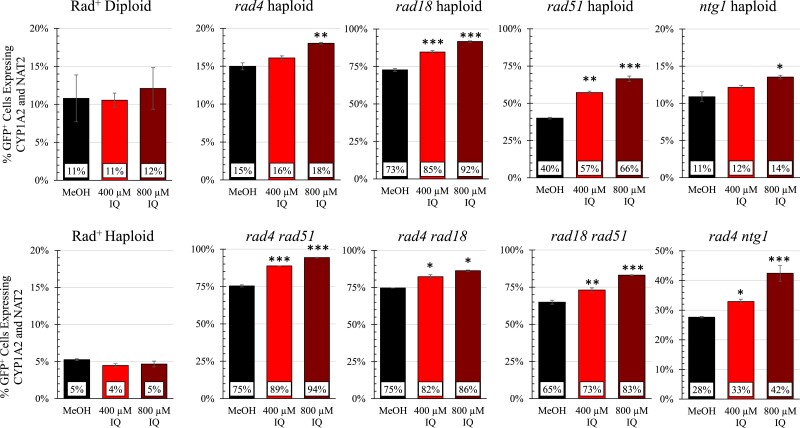
IQ toxicity in *rad4, ntg1, rad18,* and *rad51* single and double mutants. The competitive growth of selected single haploid DNA repair mutants (top row) and double mutants (bottom row) after growth in the presence of 2% MeOH, 400 µM IQ, or 800 µM IQ. Cultures were inoculated at approximately 10% of GFP-expressing wild-type cells and 90% mutant cells and were grown for 20 hours (∼10 generations). 2 × 10^4^ cells were acquired for each sample. For the Rad^+^ haploid (YB682) *N* = 8. For the Rad^+^ diploid (YB679), *N* = 12. For the other strains, *N* = 2. Error bars represent 1 standard deviation. Significance was determined by Dunnett's test and asterisks indicate *P* < 0.05 (*), *P* < 0.01 (**) and *P* < 0.001(***).

Fifty-one ORFs appeared to either confer both IQ sensitivity (m > 1, q < 0.1) or resistance (m < 1, q < 0.1). Among these were the DNA repair genes, *NTG1* and *RAD18*. Both trypan blue and competitive growth assays, however, indicate that these are validated resistance genes (Table 1). Both haploid *rad18* and *ntg1* strains also exhibit enhanced IQ sensitivity (Fig. 6), compared to the wild-type. Thus, *NTG1* and *RAD18* confer IQ resistance in both haploid and diploid strains.

### Enhanced sensitivity of haploid double mutants defective in DNA repair

We hypothesize that 1 reason we did not identify more DNA repair genes is that there are multiple repair functions for IQ-associated DNA damage. To test this hypothesis, we made *rad4  rad51*, *rad4  rad18*, *rad4  ntg1*, and *rad18  rad51* haploid double mutants. We compared the double mutants and the corresponding single mutant haploids using growth curves, competitive growth, and viability assays. Competitive growth data for these double mutants, along with the haploid single mutants, expressing CYP1A2 and NAT2 are seen in Fig. 6. IQ-associated cell death data are shown in Supplementary Table 5. Growth curves for double and single mutants are shown in Supplementary Fig. 3. Compared to respective single mutants, the IQ sensitivity was most enhanced for the *rad4  ntg1* and *rad4  rad51* double mutants. Collectively these data suggest that *NTG1* and *RAD4*, and *RAD4* and *RAD51* function in multiple DNA repair pathways for IQ-associated DNA damage.

### Human orthologs of yeast IQ resistance genes associated with cancer

The genes appearing in at least 2 screens were checked for human orthologs using the *Saccharomyces* Genome Database (SGD, https://www.yeastgenome.org). Human orthologs were then checked for disease association using Alliance of Genome Resources (https://www.alliancegenome.org) to determine if any were implicated in human cancer (Sternberg et al. 2022). Of the human orthologs of IQ resistance genes, there were 11 genes associated with colon cancer and 10 genes associated with other or multiple cancers (Table 5). This is of particular interest because these compounds are often found in cooked red meats, which is a probable human carcinogen, and a risk factor for CRC. Biallelic mutations in the human ortholog of *NTG1*, *NTHL1,* are a high-risk factor for familial adenomatous polyposis (Groden et al. 1991). The human homolog for *RAD18* has been noted to participate in multiple cancer progression stages and over-expression confers resistance to chemotherapeutic agents (Yang et al. 2018, Li et al. 2020). Alleles in other colon cancer risk-associated genes include *SET2* and *SHH3* (Habano et al. 2003, Choi et al. 2014). In addition, other genes were associated with multiple cancers, including breast and colon cancer. These include *ADE6*, which encodes phosphoribosylformylglycinamidine (FGAM) synthase, and *TEP1*, which is the yeast ortholog for the tumor suppressor gene *PTEN*.

**Table 5.** Yeast genes conferring resistance to activated IQ with human orthologs associated with cancer.

Gene*^a^*	Human*^b^*	Function	Cancer association	References
*ADE6*	**PFAS**	Phosphoribosyl formylglycinamidine synthase	Colon, liver	Elliott and Weber 1984
*ALT1*	GPT/GPT2	Alanine transaminase, involved in alanine biosynthesis, and catabolism	Liver	Rafter et al. 2012
BRE1*	RNF20	E3 ubiquitin ligase	Breast	Shema et al. 2017
*CHK1*	CHEK1	DNA damage checkpoint effector, serine/threonine kinase	Pancreatic	Okazaki et al. 2008
*CKA1*	CSNK2A1	A Ser/Thr protein kinase with roles in cell growth and proliferation	Breast	Landesman-Bollag et al. 2001
*CTS2*	**CHI3L1**	Putative chitinase	Colon	Cintin et al. 2002
*GAT1*	**GATA1/GATA6**	Transcriptional activator of nitrogen catabolite repression	Colon	Yu et al. 2019; Tsuji et al. 2014
*GNP1/MMP1*	**SLC7A5**	Glutamine permease/S-methylmethionine permease	Colon	Sakata et al. 2020
*IDP1*	IDH1	Mitochondrial NADP-specific isocitrate dehydrogenase	Multiple	Willander et al. 2014; Lee et al. 2017
JHD1*	PHF8/KDM2A	JmjC domain family histone demethylase specific for H3-K36	Lung	Dhar et al. 2014
MET13*	MTHFR	Major isozyme of methylenetetrahydrofolate reductase	Multiple	Safarinejad et al. 2011; Yuen et al. 2007
*NTG1*	**NTHL1**	Endonuclease III-like glycosylase	Colon	Broderick et al. 2017; Galick et al. 2013
*RAD18*	**RAD18**	DNA damage-bypass by error-prone and error-free mechanisms	Colon	Kanzaki et al. 2007
*SET2*	**SETD2**	H3-K36 histone methyltransferase. Regulates histone acetylation + exchange, and DNA-templated transcription	Colon	Choi et al. 2014
*SHH3*	**SDH3**	Succinate dehydrogenase	Colon	Habano et al. 2003
SPO14*	PLD2	Phospholipase D	Kidney	Zhao et al. 2000
*TEP1*	**PTEN**	Homolog of human tumor suppressor gene PTEN. No demonstrated inositol lipid phosphatase activity	Multiple, including colon	Lynch et al. 1997; Roseweir et al. 2016; Jaegar et al. 2011
YVC1*	**TRPV3**	Vacuolar cation channel	Colon	Hoeft et al. 2010

GPT, Glutamic--Pyruvic Transaminase.

^*a*^Asterisk (*) indicates genes that have not been independently validated.

^*b*^Bolded genes are associated with colorectal cancer.

## Discussion

IQ is a class 2A and rodent carcinogen, present in charred meats in cigarette smoke. In this study, we profiled the yeast genome for IQ resistance by screening a “humanized” yeast diploid deletion library that expresses both CYP1A2 and NAT2. We identified 424 ORFs that appeared in at least 2 of 4 independent screens of the “humanized” library, and validated 82 using either growth curves, competitive growth assays, or viability assays. Major ontology groups included DNA binding, phosphatase, and ribosomal protein genes, while protein complexes included HIR and CK2. Human orthologs of 11 yeast genes are associated with colon cancer. We speculate that the colon cancer risk associated with these genetic factors could be increased by exposure to HAAs.

Several conclusions can be drawn from our studies screening the “humanized” and original yeast library. First, both screens identified ribosomal protein genes as resistance genes, thus suggesting that IQ targets ribosomes, and by extension, protein synthesis. Second, prominent among DNA repair genes were postreplication repair genes and *NTG1*, while NER genes were less prominent. Third, a comparison of the toxicity of IQ with other toxins underscores the role of several important genes and complexes involved in DNA damage and genotoxin tolerance, including *RAD18* and the *CK2* complex.

These conclusions were based on profiling the yeast genome for IQ resistance using a “humanized” library that expresses both CYP1A2 and NAT2. As expected, more ORFs were identified in the screen using a higher concentration of IQ in the “humanized” library and most genes identified at the lower concentration were also identified at the higher concentration. Nonetheless, there were several limitations to this study. First, many DNA repair-deficient strains were not detected even after exposure for 5 generations; a similar limitation was observed in a screen of a “humanized” library for AFB_1_ resistance and was partially attributed to CYP1A2 expression, which confers an enhanced growth disadvantage (St John et al. 2020). For example, *mre11, xrs2,* and *rad52* strains were not detected at all in the pooled samples, even without treatment. Second, genome profiling necessitated relatively high IQ concentrations that likely conferred toxicity by additional nongenotoxic mechanisms. While IQ sensitivity is enhanced in strains expressing CYP1A2 and NAT2, we do not know the exact proportion or amounts of activated IQ metabolites and all their associated targets.

Among the ribosomal protein genes required for resistance to both “activated” IQ and IQ, the oxidative stress response gene, *RPL22A* (Chan et al. 2012), is present in both sets, while genes that function in rRNA export from the nucleus were only present among “activated” IQ resistance genes. Both IQ and activated IQ resistance genes encode rRNA proteins located in the large and small ribosomal subunits; however, there are more “activated” IQ resistance genes that function in a wider set of RNA translation functions and ribosomal protein transport (Supplementary Table 4 and Fig. 1). This set included genes that facilitate RNA translation, such as *DEG1*, modify tRNAs, such as *PUS7*, *MOD5*, and *DUS1*, and import ribosomal proteins into the nucleus, such as *SYO1*. Taken together, these data suggest that in comparison to resistance to IQ, resistance to activated IQ requires a larger set of genes involved in protein translation and rRNA transport.

Many ribosomal protein genes that confer IQ resistance also confer resistance to other toxins. Typical cellular reactions to multiple stresses involve downregulation of ribosomal protein gene transcription (Warner 1999; Gasch et al. 2000), which may render ribosomal protein mutants sensitive to mycotoxins and antifungal agents. Ribosome quality control is a central feature of resistance to multiple toxins, including trichothecene mycotoxins and cycloheximide (Kugler et al. 2016). Nonetheless, major cycloheximide resistance genes, *RPL41* and *RPL29* (Käufer et al. 1983; Dehoux et al. 1993; Bae et al. 2018), did not appear in our screen for IQ resistance but instead appeared to confer IQ sensitivity (Supplementary Tables 4 and 7).

The notion that activated IQ targets proteins is supported by observations that human serum albumin (SA)Cys^34^ readily reacts with HAAs (Turesky and Le Marchand 2011; Bellamri et al. 2021) and that IQ reacts with heme-containing proteins (Turesky et al. 1987), suggesting that similar reactions could occur with yeast proteins. IQ-associated protein modification may explain why ammonia, amino acid metabolism, and protein synthesis were heavily represented among HAA resistance genes (Supplementary Table 4). Since protein modification genes (*ALG3, ALG5, ALG6, ALG8, ALG12*) were also identified in the IQ screen of the original deletion library (Supplementary Table 7 and Fig. 2), we presume that some cellular damage could also occur by IQ exposure per se or by IQ metabolites generated by endogenous yeast enzymes.

Our data supports the notion that replication bypass functions in IQ resistance, as was suggested by in vitro experiments using oligonucleotides containing IQ-N^2^ Gua adducts (Stavros 2014). DNA repair genes that conferred IQ resistance were largely confined to those participating in postreplication repair. These included *RAD18*, *REV7*, and *UBC13*, which are involved in DNA damage tolerance pathways that bypass DNA damage by error-prone polymerases (for review, see Xu et al. 2015). We also identified *MPH1*, a gene whose helicase function resolves stalled DNA structures, and which functions in error-free repair (Schürer et al. 2004).

*RAD18* and *REV7*, which confer IQ resistance, are also common resistance genes for multiple cross-linking agents and agents that cause DNA adducts. These include trichloroethylene (De la Rosa et al. 2017) and AFB_1_ (St. John et al. 2020), as well as chemotherapeutic drug derivatives, such as cisplatin, oxaliplatin, and mitomycin C (Wu et al. 2004). *RAD18* may also function in error-free mechanisms that efficiently bypass N^2^-Gua adducts (Washington et al. 2004). The contribution of both error-free and error-prone mechanisms in bypassing IQ-associated DNA adducts will require additional studies.

Other protein kinases and phosphatases may have secondary roles in conferring tolerance to IQ-associated damage. Both the CK2 complex genes and *PSY2* confer tolerance to multiple DNA-damaging agents (St. John et al. 2020). *PSY2* encodes a phosphatase that deactivates Rad53 after exposure to a subset of DNA-damaging agents (O’Neill et al. 2007), while the CK2 complex is required for toleration of irreparable double-strand breaks (Toczyski et al. 1997).

In the repair of IQ-N^2^ Gua or oxidized DNA bases, NER may not serve as a primary DNA repair mechanism, as suggested by Stavros et al. (2014). This may partially explain why *rad4* mutants exhibited modest IQ sensitivity, while the double *rad4  rad51* and *rad4  ntg1* mutants exhibit enhanced IQ sensitivities. A synergistic relationship between *NTG1* and *RAD4* has been previously observed for oxidative stress resistance (Gellon et al. 2001). Further studies are necessary to determine whether the IQ-N^2^ Gua adduct can be recognized by NER enzymes.

Eleven genes of the 424 ORFs that confer IQ resistance are associated with colon cancer (Table 4). Alleles mapping in human orthologs *RAD18* and *NTHL1*, increase the risk of colon cancer. *RAD18 Arg302Gln* increases the colon cancer risk in Japanese patients (Kanzaki et al. 2007), while biallelic mutations in *NTHL1*, result in *NTHL1*-associated polyposis (Weren et al. 2015). Both *NTG1* and *NTHL1* encode bifunctional glycosylases, containing both glycosylase and lyase activity. *NTG1* is noted to function in a redundant pathway to reduce G:C to T:A transversions, while in humans, formamido-pyrimidine residues, but not 8-oxyG, are recognized by NTHL1 (Das et al. 2020). Decreased activity is correlated with a mutation signature 30, where C is converted to T, while increased activity correlates with sequestration of the NER gene *XPG*, the mammalian *RAD2* ortholog. These studies further underscore the primary role of base excision repair and a secondary role of NER in repairing oxidative DNA damage.

In summary, we profiled the yeast genome for IQ resistance using 2 different yeast libraries; 1 which was capable of bioactivation and the other which was not. Similarities between the IQ resistance genes indicated that protein was an IQ target, while in the “humanized” library, we identified DNA damage tolerance genes and *NTG1*. Considering that human orthologs *NTHL1* and *RAD18* are risk factors for colon cancer, our results provoke further studies suggesting that polymorphic *NTHL1* alleles may confer HAA sensitivities.

## Data Availability

All yeast strains and plasmids are available upon request. Specific yeast strains used in this study are listed in Supplementary Table 1. Next-generation sequencing (NGS) data of barcodes can be found at gene expression omnibus (https://www.ncbi.nlm.nih.gov/geo/query/acc.cgi? acc=GSE216310). Eight supplementary tables, 3 supplementary figures, and 2 supplementary files can be accessed on figshare: https://doi.org/10.25387/g3.24069477. Supplementary File S1 contains the R code for processing barcode counts. Supplementary File S2 contains the normalized counts for each strain in each screen, as well as the processed differential abundance data. Supplementary Fig. S1 shows the interaction of all proteins encoded by the IQ resistance genes identified in the “humanized” yeast deletion collection under any condition. Supplementary Fig. S2a shows all proteins encoded by the IQ resistance genes in the original yeast deletion collection, and Supplementary Fig. S2b shows the IQ resistance genes appearing twice in screens with the original collection. Supplementary Figure S3 shows the growth curve data for the “humanized” double mutants and corresponding “humanized” single mutants, as well as the “humanized” WT haploid and diploid controls. Supplementary Table S1 contains the list of specific strains used in this study. Supplementary Table S2 contains the oligos used for high-throughput sequencing and multiplexing. Supplementary Table S3 contains mutagenicity data from the Ames assay. Supplementary Table S4 contains the full list of IQ resistance genes identified from the “humanized” yeast deletion collection in either screen. Supplementary Table S5 contains the list of IQ resistance genes that were independently validated. Supplementary Table S6 contains GO terms for genes identified in at least two screens of the “humanized” library. Supplementary Table S7 contains the full list of IQ resistance genes identified in the original yeast deletion collection. Supplementary Table S8 contains the list of GO terms for IQ resistance genes which confer resistance with or without bioactivation of IQ.

## References

[jkad219-B1] Aeschbacher HU, Turesky RJ. 1991. Mammalian cell mutagenicity and metabolism of heterocyclic aromatic amines. Mutat Res. 259(3–4):235–250. doi:10.1016/0165-1218(91)90120-B. 2017210

[jkad219-B2] Aggarwal N, Donald ND, Malik S, Selvendran SS, McPhail MJW, Monahan KJ. 2017. The association of low-penetrance variants in DNA repair genes with colorectal cancer: a systematic review and meta-analysis. Clin Transl Gastroenterol. 8(7):e109. doi:10.1038/ctg.2017.35. 28749454 PMC5539343

[jkad219-B3] Al-Tassan N, Chmiel NH, Maynard J, Fleming N, Livingston AL, Williams GT, Hodges AK, Davies DR, David SS, Sampson JR, et al 2002. Inherited variants of *MYH* associated with somatic G:c→T:a mutations in colorectal tumors. Nat Genet. 30(2):227–232. doi:10.1038/ng828. 11818965

[jkad219-B4] Ausubel FM . 2003. Current Protocols in Molecular Biology. New York Greene Pub. Associates. p. 1.7.1–1.7.15.

[jkad219-B5] Aykan NF . 2015. Red meat and colorectal cancer. Oncol Rev. 9(1):288. doi:10.4081/oncol.2015.288. 26779313 PMC4698595

[jkad219-B6] Bae JH, Sung BH, Sohn JH. 2018. Site saturation mutagenesis of ribosomal protein L42 at 56th residue and application as a consecutive selection marker for cycloheximide resistance in yeast. FEMS Microbiol Lett. 365(8):1–5. doi:10.1093/femsle/fny066. 29566228

[jkad219-B7] Bellamri M, Walmsley SJ, Turesky RJ. 2021. Metabolism and biomarkers of heterocyclic aromatic amines in humans. Genes Environ. 43(1):29. doi:10.1186/s41021-021-00200-7. 34271992 PMC8284014

[jkad219-B8] Boyle EI, Weng S, Gollub J, Jin H, Botstein D, Cherry JM, Sherlock G. 2004. GO::TermFinder—open source software for accessing gene ontology information and finding significantly enriched gene ontology terms associated with a list of genes. Bioinformatics. 20(18):3710–3715. doi:10.1093/bioinformatics/bth456. 15297299 PMC3037731

[jkad219-B9] Brachmann CB, Davies A, Cost GJ, Caputo E, Li J, Hieter P, Boeke JD. 1998. Designer deletion strains derived from *Saccharomyces cerevisiae* S288C: a useful set of strains and plasmids for PCR-mediated gene disruption and other applications. Yeast. 14(2):115–132. doi:10.1002/(SICI)1097-0061(19980130)14:2<115::AID-YEA204>3.0.CO;2-2. 9483801

[jkad219-B10] Broderick P, Dobbins SE, Chubb D, Kinnersley B, Dunlop MG, Tomlinson I, Houlston RS. 2017. Validation of recently proposed colorectal cancer susceptibility gene variants in an analysis of families and patients—a systematic review. Gastroenterology. 152(1):75–77.e4. doi:10.1053/j.gastro.2016.09.041. 27713038 PMC5860724

[jkad219-B11] Burke D, Dawson D, Stearns T, Laboratory CSH. 2000. Methods in Yeast Genetics: A Cold Spring Harbor Laboratory Course Manual. New York: Cold Spring Harbor Laboratory Press.

[jkad219-B12] Butler LM, Millikan RC, Sinha R, Keku TO, Winkel S, Harlan B, Eaton A, Gammon MD, Sandler RS. 2008. Modification by N-acetyltransferase 1 genotype on the association between dietary heterocyclic amines and colon cancer in a multiethnic study. Mutation Research. 638(1–2):162–174. doi:10.1016/j.mrfmmm.2007.10.002. 18022202 PMC2234436

[jkad219-B13] Carvalho AM, Miranda AM, Santos FA, Loureiro APM, Fisberg RM, Marchioni DM. 2015. High intake of heterocyclic amines from meat is associated with oxidative stress. Br J Nutr. 113(8):1301–1307. doi:10.1017/S0007114515000628. 25812604

[jkad219-B14] Chan CTY, Pang YLJ, Deng W, Babu IR, Dyavaiah M, Begley TJ, Dedon PC. 2012. Reprogramming of tRNA modifications controls the oxidative stress response by codon-biased translation of proteins. Nat Commun. 3(1):937. doi:10.1038/ncomms1938. 22760636 PMC3535174

[jkad219-B15] Chen Y, Sanchez Y. 2004. Chk1 in the DNA damage response: conserved roles from yeasts to mammals. DNA Repair (Amst). 3(8–9):1025–1032. doi:10.1016/j.dnarep.2004.03.003. 15279789

[jkad219-B16] Cherry JM, Hong EL, Amundsen C, Balakrishnan R, Binkley G, Chan ET, Christie KR, Costanzo MC, Dwight SS, Engel SR, et al 2012. Saccharomyces Genome Database: the genomics resource of budding yeast. Nucleic Acids Res. 40(D1):D700–D705. doi:10.1093/nar/gkr1029. 22110037 PMC3245034

[jkad219-B17] Choi YJ, Oh HR, Choi MR, Gwak M, An CH, Chung YJ, Yoo NJ, Lee SH. 2014. Frameshift mutation of a histone methylation-related gene *SETD1B* and its regional heterogeneity in gastric and colorectal cancers with high microsatellite instability. Hum Pathol. 45(8):1674–1681. doi:10.1016/j.humpath.2014.04.013. 24925220

[jkad219-B18] Cintin C, Johansen JS, Christensen IJ, Price PA, Sørensen S, Nielsen HJ. 2002. High serum YKL-40 level after surgery for colorectal carcinoma is related to short survival. Cancer. 95(2):267–274. doi:10.1002/cncr.10644. 12124825

[jkad219-B19] Das L, Quintana VG, Sweasy JB. 2020. NTHL1 In genomic integrity, aging and cancer. DNA Repair (Amst). 93:102920. doi:10.1016/j.dnarep.2020.102920. 33087284 PMC7748067

[jkad219-B20] de Carvalho AM, Carioca AAF, Fisberg RM, Qi L, Marchioni DM. 2016. Joint association of fruit, vegetable, and heterocyclic amine intake with DNA damage levels in a general population. Nutrition. 32(2):260–264. doi:10.1016/j.nut.2015.08.018. 26530455

[jkad219-B21] Dehoux P, Davies J, Cannon M. 1993. Natural cycloheximide resistance in yeast: the role of ribosomal protein L41. Eur J Biochem. 213(2):841–848. doi:10.1111/j.1432-1033.1993.tb17827.x. 8477753

[jkad219-B22] De La Rosa VY, Asfaha J, Fasullo M, Loguinov A, Li P, Moore LE, Rothman N, Nakamura J, Swenberg JA, Scelo G, et al 2017. High-throughput functional genomics identifies modulators of TCE metabolite genotoxicity and candidate susceptibility genes. Toxicol Sci. 160(1):111–120. doi:10.1093/toxsci/kfx159. 28973557 PMC5837773

[jkad219-B23] Dhar SS, Alam H, Li N, Wagner KW, Chung J, Ahn YW, Lee MG. 2014. Transcriptional repression of histone deacetylase 3 by the histone demethylase KDM2A is coupled to tumorigenicity of lung cancer cells. J Biol Chem. 289(11):7483–7496. doi:10.1074/jbc.M113.521625. 24482232 PMC3953262

[jkad219-B24] Elliott WL, Weber G. 1984. Proliferation-linked increase in phosphoribosylformylglycinamidine synthetase activity (EC 6.3.5.3)1. Cancer Res. 44(6):2430–2434. 6722784

[jkad219-B25] Fasullo M, Chen Y, Bortcosh W, Sun M, Egner PA. 2010. Aflatoxin B1-associated DNA adducts stall S phase and stimulate Rad51 foci in *Saccharomyces cerevisiae*. J Nucleic Acids. 2010:456487. doi:10.4061/2010/456487. 21151658 PMC2997344

[jkad219-B26] Fasullo M, Giallanza P, Dong Z, Cera C, Bennett T. 2001. *Saccharomyces cerevisiae* rad51 mutants are defective in DNA damage-associated sister chromatid exchanges but exhibit increased rates of homology-directed translocations. Genetics. 158(3):959–972. doi:10.1093/genetics/158.3.959. 11454747 PMC1461715

[jkad219-B27] Fasullo M, Smith A, Egner P, Cera C. 2014. Activation of aflatoxin B1 by expression of human CYP1A2 polymorphisms in *Saccharomyces cerevisiae*. Mutat Res Genet Toxicol Environ Mutagen. 761:18–26. doi:10.1016/j.mrgentox.2014.01.009. 24472830 PMC4707934

[jkad219-B28] Foiani M, Marini F, Gamba D, Lucchini G, Plevani P. 1994. The B subunit of the DNA polymerase alpha-primase complex in *Saccharomyces cerevisiae* executes an essential function at the initial stage of DNA replication. Mol Cell Biol. 14(2):923–933. doi:10.1128/mcb.14.2.923-933.1994. 8289832 PMC358447

[jkad219-B29] Fretland AJ, Leff MA, Doll MA, Hein DW. 2001. Functional characterization of human N-acetyltransferase 2 (NAT2) single nucleotide polymorphisms. Pharmacogenetics. 11(3):207–215. doi:10.1097/00008571-200104000-00004. 11337936

[jkad219-B30] Galick HA, Kathe S, Liu M, Robey-Bond S, Kidane D, Wallace SS, Sweasy JB. 2013. Germ-line variant of human NTH1 DNA glycosylase induces genomic instability and cellular transformation. Proc Natl Acad Sci U S A. 110(35):14314–14319. doi:10.1073/pnas.1306752110. 23940330 PMC3761600

[jkad219-B31] Gasch AP, Spellman PT, Kao CM, Carmel-Harel O, Eisen MB, Storz G, Botstein D, Brown PO. 2000. Genomic expression programs in the response of yeast cells to environmental changes. Mol Biol Cell. 11(12):4241–4257. doi:10.1091/mbc.11.12.4241. 11102521 PMC15070

[jkad219-B32] Gellon L, Barbey R, Auffret Van der Kemp P, Thomas D, Boiteux S. 2001. Synergism between base excision repair, mediated by the DNA glycosylases ntg1 and ntg2, and nucleotide excision repair in the removal of oxidatively damaged DNA bases in Saccharomyces cerevisiae. Mol Genet Genomics. 265(6):1087–1096. doi:10.1007/s004380100507. 11523781

[jkad219-B33] Giaever G, Chu AM, Ni L, Connelly C, Riles L, Véronneau S, Dow S, Lucau-Danila A, Anderson K, André B, et al 2002. Functional profiling of the *Saccharomyces cerevisiae* genome. Nature. 418(6896):387–391. doi:10.1038/nature00935. 12140549

[jkad219-B34] Giaever G, Nislow C. 2014. The yeast deletion collection: a decade of functional genomics. Genetics. 197(2):451–465. doi:10.1534/genetics.114.161620. 24939991 PMC4063906

[jkad219-B35] Gongora VM, Matthes KL, Castaño PR, Linseisen J, Rohrmann S. 2019. Dietary heterocyclic amine intake and colorectal adenoma risk: a systematic review and meta-analysis. Cancer Epidemiol Biomarkers Prev. 28(1):99–109. doi:10.1158/1055-9965.EPI-17-1017. 30275115

[jkad219-B36] Grant DM, Hughes NC, Janezic SA, Goodfellow GH, Chen HJ, Gaedigk A, Yu VL, Grewal R. 1997. Human acetyltransferase polymorphisms. Mutat Res. 376(1–2):61–70. doi:10.1016/S0027-5107(97)00026-2. 9202739

[jkad219-B37] Grant DM, Josephy PD, Lord HL, Morrison LD. 1992. *Salmonella typhimurium* strains expressing human arylamine JV-acetyltransferases: metabolism and mutagenic activation of aromatic amines. Cancer Res. 52(14):3961–3964. 1617672

[jkad219-B38] Green MR, Sambrook J. 2019. Isolation of DNA fragments from polyacrylamide gels by the crush and soak method. Cold Spring Harb Protoc. 2019(2). doi:10.1101/pdb.prot100479. 30710028

[jkad219-B39] Groden J, Thliveris A, Samowitz W, Carlson M, Gelbert L, Albertsen H, Joslyn G, Stevens J, Spirio L, Robertson M, et al 1991. Identification and characterization of the familial adenomatous polyposis coli gene. Cell. 66(3):589–600. doi:10.1016/0092-8674(81)90021-0. 1651174

[jkad219-B40] Habano W, Sugai T, Nakamura SI, Uesugi N, Higuchi T, Terashima M, Horiuchi S. 2003. Reduced expression and loss of heterozygosity of the *SDHD* gene in colorectal and gastric cancer. Oncol Rep. 10(5):1375–1380. doi:10.3892/or.10.5.1375. 12883710

[jkad219-B41] Hoeft B, Linseisen J, Beckmann L, Müller-Decker K, Canzian F, Hüsing A, Kaaks R, Vogel U, Jakobsen MU, Overvad K, et al 2010. Polymorphisms in fatty acid metabolism-related genes are associated with colorectal cancer risk. Carcinogenesis. 31(3):466–472. doi:10.1093/carcin/bgp325. 20042636

[jkad219-B42] Hoffman CS, Winston F. 1987. A ten-minute DNA preparation from yeast efficiently releases autonomous plasmids for transformation of *Escherichia coli*. Gene. 57(2–3):267–272. doi:10.1016/0378-1119(87)90131-4. 3319781

[jkad219-B43] IARC Working Group on the Evaluation of Carcinogenic Risks to Humans . 1993. Some Naturally Occurring Substances: Food Items and Constituents, Heterocyclic Aromatic Amines and Mycotoxins. UK: World Health Organization, International Agency for Research on Cancer. p. 165–195.

[jkad219-B44] Jaeger S, Iliopoulos D, Pothoulakis C. 2011. Cancer in humans and mice. Gastroenterology. 141(1):1–19. doi:10.1053/j.gastro.2011.07.038.Neurotensin. 21806946 PMC4442678

[jkad219-B45] Jägerstad M, Skog K, Grivas S, Olsson K. 1991. Formation of heterocyclic amines using model systems. Mutat Res. 259(3–4):219–233. doi:10.1016/0165-1218(91)90119-7. 2017209

[jkad219-B46] Jo WJ, Loguinov A, Wintz H, Chang M, Smith AH, Kalman D, Zhang L, Smith MT, Vulpe CD. 2009. Comparative functional genomic analysis identifies distinct and overlapping sets of genes required for resistance to monomethylarsonous acid (MMAIII) and arsenite (AsIII) in yeast. Toxicol Sci. 111(2):424–436. doi:10.1093/toxsci/kfp162. 19635755 PMC2742584

[jkad219-B47] Josephy PD, DeBruin LS, Lord HL, Oak JN, Evans DH, Guo Z, Dong MS, Guengerich FP. 1995. Bioactivation of aromatic amines by recombinant human cytochrome P4501A2 expressed in Ames tester strain Bacteria: a substitute for activation by mammalian tissue preparations. Cancer Res. 55(4):799–802. 7850792

[jkad219-B48] Kachroo AH, Laurent JM, Yellman CM, Meyer AG, Wilke CO, Marcotte EM. 2015. Evolution. Systematic humanization of yeast genes reveals conserved functions and genetic modularity. Science. 348(6237):921–925. doi:10.1126/science.aaa0769. 25999509 PMC4718922

[jkad219-B49] Kanzaki H, Ouchida M, Hanafusa H, Sakai A, Yamamoto H, Suzuki H, Yano M, Aoe M, Imai K, Date H, et al 2007. Single nucleotide polymorphism in the *RAD18* gene and risk of colorectal cancer in the Japanese population. Oncol Rep. 18(5):1171–1175. doi:10.3892/or.18.5.1171. 17914568

[jkad219-B50] Kasai H, Nishimura S, Wakabayashi K, Nagao M, Sugimura T. 1980. Chemical synthesis of 2-amino-3-methylimidazo [4,5-f] quinoline (IQ), a potent mutagen isolated from broiled fish. Proc Japan Acad. 56(6):382–384. doi:10.2183/pjab.56.382.

[jkad219-B51] Kato R . 1986. Metabolic activation of mutagenic heterocyclic aromatic amines from protein pyrolysates. CRC Crit Rev Toxicol. 16(4):307–348. doi:10.3109/10408448609037466. 3519087

[jkad219-B52] Käufer NF, Fried HM, Schwindinger WF, Jasin M, Warner JR. 1983. Cycloheximide resistance in yeast: the gene and its protein. Nucleic Acids Res. 11(10):3123–3135. doi:10.1093/nar/11.10.3123. 6304624 PMC325953

[jkad219-B53] Kim D, Guengerich FP. 2005. Cytochrome P450 activation of arylamines and heterocyclic amines. Annu Rev Pharmacol Toxicol. 45(1):27–49. doi:10.1146/annurev.pharmtox.45.120403.100010. 15822170

[jkad219-B54] Knasmüller S, Schwab CE, Land SJ, Wang C, Sanyal R, Kundi M, Parzefall W, Darroudi F. 1999. Genotoxic effects of heterocyclic aromatic amines in human derived hepatoma (HepG2) cells. Mutagenesis. 14(6):533–540. doi:10.1093/mutage/14.6.533. 10567027

[jkad219-B55] Kugler KG, Jandric Z, Beyer R, Klopf E, Glaser W, Lemmens M, Shams M, Mayer K, Adam G, Schüller C. 2016. Ribosome quality control is a central protection mechanism for yeast exposed to deoxynivalenol and trichothecin. BMC Genomics. 17(1):417. doi:10.1186/s12864-016-2718-y. 27245696 PMC4888481

[jkad219-B56] Landesman-Bollag E, Romieu-Mourez R, Song DH, Sonenshein GE, Cardiff RD, Seldin DC. 2001. Protein kinase CK2 in mammary gland tumorigenesis. Oncogene. 20(25):3247–3257. doi:10.1038/sj.onc.1204411. 11423974

[jkad219-B57] Lee JH, Shin DH, Park WY, Shin N, Kim A, Lee HJ, Kim YK, Choi KU, Kim JY, Il YY, et al 2017. *IDH1* R132c mutation is detected in clear cell hepatocellular carcinoma by pyrosequencing. World J Surg Oncol. 15(1):82. doi:10.1186/s12957-017-1144-1. 28403884 PMC5389153

[jkad219-B58] Le Marchand L . 2021. The role of heterocyclic aromatic amines in colorectal cancer: the evidence from epidemiologic studies. Genes Environ. 43(1):20. doi:10.1186/s41021-021-00197-z. 34099058 PMC8183058

[jkad219-B59] Lesser CF, Guthrie C. 1993. Mutational analysis of pre-mRNA splicing *in Saccharomyces cerevisiae* using a sensitive new reporter gene, *CUPl*. Genetics. 133(4):851–863. doi:10.1093/genetics/133.4.851. 8462846 PMC1205405

[jkad219-B60] Li P, He C, Gao A, Yan X, Xia X, Zhou J, Wu J. 2020. RAD18 Promotes colorectal cancer metastasis by activating the epithelial-mesenchymal transition pathway. Oncol Rep. 44(1):213–223. doi:10.3892/or.2020.7590. 32319669 PMC7251712

[jkad219-B61] Liesche J, Marek M, Günther-Pomorski T. 2015. Cell wall staining with trypan blue enables quantitative analysis of morphological changes in yeast cells. Front Microbiol. 6:107. doi:10.3389/fmicb.2015.00107. 25717323 PMC4324143

[jkad219-B62] Lilla C, Verla-Tebit E, Risch A, Jäger B, Hoffmeister M, Brenner H, Chang-Claude J. 2006. Effect of *NAT1* and *NAT2* genetic polymorphisms on colorectal cancer risk associated with exposure to tobacco smoke and meat consumption. Cancer Epidemiol Biomarkers Prev. 15(1):99–107. doi:10.1158/1055-9965.EPI-05-0618. 16434594

[jkad219-B63] Linseisen J, Kesse E, Slimani N, Bueno-de-Mesquita H, Ocké M, Skeie G, Kumle M, Iraeta MD, Gómez PM, Janzon L, et al 2002. Meat consumption in the European Prospective Investigation into Cancer and Nutrition (EPIC) cohorts: results from 24-hour dietary recalls. Public Health Nutr. 5(6b):1243–1258. doi:10.1079/phn2002402. 12639230

[jkad219-B64] Lynch ED, Ostermeyer EA, Lee MK, Arena JF, Ji H, Dann J, Swisshelm K, Suchard D, MacLeod PM, Kvinnsland S, et al 1997. Inherited mutations in PTEN that are associated with breast cancer, Cowden disease, and juvenile polyposis. Am J Hum Genet. 61(6):1254–1260. doi:10.1086/301639. 9399897 PMC1716102

[jkad219-B65] Maron DM, Ames BN. 1983. Revised methods for the Salmonella mutagenicity test. Mutat Res. 113(3–4):173–215. doi:10.1016/0165-1161(83)90010-9. 6341825

[jkad219-B66] Matejcic M, Shaban HA, Quintana MW, Schumacher FR, Edlund CK, Naghi L, Pai RK, Haile RW, Joan Levine A, Buchanan DD, et al 2021. Rare variants in the DNA repair pathway and the risk of colorectal cancer. Cancer Epidemiol Biomarker Prev. 30(5):895–903. doi:10.1158/1055-9965.EPI-20-1457. PMC8102340 33627384

[jkad219-B67] Metry KJ, Neale JR, Doll MA, Howarth AL, States JC, McGregor WG, Pierce WM, Hein DW. 2010. Effect of rapid human N-acetyltransferase 2 haplotype on DNA damage and mutagenesis induced by 2-amino-3-methylimidazo-[4,5-f]quinoline (IQ) and 2-amino-3,8-dimethylimidazo-[4,5-f]quinoxaline (MeIQx). Mutat Res. 684(1–2):66–73. doi:10.1016/j.mrfmmm.2009.12.001. 20004212 PMC2820402

[jkad219-B68] Nohmi T, Watanabe M. 2021. Mutagenicity of carcinogenic heterocyclic amines in *Salmonella typhimurium* YG strains and transgenic rodents including *gpt* delta. Genes Environ. 43(1):1–24. doi:10.1186/s41021-021-00207-0. 34526143 PMC8444484

[jkad219-B69] North M, Steffen J, Loguinov A V, Zimmerman GR, Vulpe CD, Eide DJ. 2012. Genome-wide functional profiling identifies genes and processes important for zinc-limited growth of *Saccharomyces cerevisiae*. PLoS Genet. 8(6):e1002699. doi:10.1371/journal.pgen.1002699. 22685415 PMC3369956

[jkad219-B70] Nöthlings U, Yamamoto JF, Wilkens LR, Murphy SP, Park SY, Henderson BE, Kolonel LN, Le Marchand L. 2009. Meat and heterocyclic amine intake, smoking, NAT1 and NAT2 polymorphisms, and colorectal cancer risk in the multiethnic cohort study. Cancer Epidemiol Biomarker Prev. 18(7):2098–2106. doi:10.1158/1055-9965.EPI-08-1218. PMC2771770 19549810

[jkad219-B71] O’Brien KP, Remm M, Sonnhammer ELL. 2005. Inparanoid: a comprehensive database of eukaryotic orthologs. Nucleic Acids Res. 33(Database issue):D476–D480. doi:10.1093/nar/gki107. 15608241 PMC540061

[jkad219-B72] Okazaki T, Jiao L, Chang P, Evans DB, Abbruzzese JL, Li D. 2008. Single-nucleotide polymorphisms of DNA damage response genes are associated with overall survival in patients with pancreatic cancer. Clin Cancer Res. 14(7):2042–2048. doi:10.1158/1078-0432.CCR-07-1520. 18381943 PMC2423806

[jkad219-B73] Olalekan Adeyeye SA, Ashaolu TJ. 2021. Heterocyclic amine formation and mitigation in processed meat and meat products: a mini-review. J Food Prot. 84(11):1868–1877. doi:10.4315/JFP-20-471. 33956955

[jkad219-B74] O’Neill BM, Szyjka SJ, Lis ET, Bailey AO, Yates JR, Aparicio OM, Romesberg FE. 2007. Pph3-Psy2 is a phosphatase complex required for Rad53 dephosphorylation and replication fork restart during recovery from DNA damage. Proc Natl Acad Sci U S A. 104(22):9290–9295. doi:10.1073/pnas.0703252104. 17517611 PMC1890487

[jkad219-B75] Paladino G, Weibel B, Sengstag C. 1999. Heterocyclic aromatic amines efficiently induce mitotic recombination in metabolically competent *Saccharomyces cerevisiae* strains. Carcinogenesis. 20(11):2143–2152. doi:10.1093/carcin/20.11.2143. 10545418

[jkad219-B76] Pan J, Chi P, Lu X, Xu Z. 2012. Genetic polymorphisms in translesion synthesis genes are associated with colorectal cancer risk and metastasis in Han Chinese. Gene. 504(2):151–155. doi:10.1016/j.gene.2012.05.042. 22652275

[jkad219-B77] Pezdirc M, Žegura B, Filipič M. 2013. Genotoxicity and induction of DNA damage responsive genes by food-borne heterocyclic aromatic amines in human hepatoma HepG2 cells. Food Chem Toxicol. 59:386–394. doi:10.1016/j.fct.2013.06.030. 23810796

[jkad219-B78] Pfau W, Martin FL, Cole KJ, Venitt S, Phillips DH, Grover PL, Marquardt H. 1999. Heterocyclic aromatic amines induce DNA strand breaks and cell transformation. Carcinogenesis. 20(4):545–551. doi:10.1093/carcin/20.4.545. 10223180

[jkad219-B79] Pompon D, Louerat B, Bronine A, Urban P. 1996. Yeast expression of animal and plant P450s in optimized redox environments. Methods Enzymol. 272:51–64. doi:10.1016/s0076-6879(96)72008-6. 8791762

[jkad219-B80] Prakash R, Satory D, Dray E, Papusha A, Scheller J, Kramer W, Krejci L, Klein H, Haber JE, Sung P, et al 2009. Yeast Mphl helicase dissociates Rad51-made D-loops: implications for crossover control in mitotic recombination. Genes Dev. 23(1):67–79. doi:10.1101/gad.1737809. 19136626 PMC2632165

[jkad219-B81] Probst-hensch NM, Han C, Lin BK, Haile RW, Ingles SA, Longnecker MP, Lee DB, Sakamoto GT, Frank HD, Lee ER, et al 1995. Acetylation polymorphism and prevalence of colorectal adenomas. Cancer Res. 55(10):2017–2020. 7743494

[jkad219-B82] Rafter I, Gråberg T, Kotronen A, Strömmer L, Mattson CM, Kim RW, Ehrenborg E, Andersson HB, Yki-Järvinen H, Schuppe-Koistinen I, et al 2012. Isoform-specific alanine aminotransferase measurement can distinguish hepatic from extrahepatic injury in humans. Int J Mol Med. 30(5):1241–1249. doi:10.3892/ijmm.2012.1106. 22922605

[jkad219-B83] Robinson DG, Chen W, Storey JD, Gresham D. 2014. Design and analysis of bar-seq experiments. G3 (Bethesda). 4(1):11–18. doi:10.1534/g3.113.008565. 24192834 PMC3887526

[jkad219-B84] Roseweir AK, Powell AGMT, Bennett L, Van Wyk HC, Park J, McMillan DC, Horgan PG, Edwards J. 2016. Relationship between tumour PTEN/Akt/COX-2 expression, inflammatory response and survival in patients with colorectal cancer. Oncotarget. 7(43):70601–70612. doi:10.18632/oncotarget.12134. 27661110 PMC5342577

[jkad219-B85] Safarinejad MR, Shafiei N, Safarinejad S. 2011. Genetic susceptibility of methylenetetrahydrofolate reductase (MTHFR) gene C677T, A1298C, and G1793A polymorphisms with risk for bladder transitional cell carcinoma in men. Med Oncol. 28(S1):398–412. doi:10.1007/s12032-010-9723-9. 21046286

[jkad219-B86] Sakata T, Hana K, Mikami T, Yoshida T, Endou H, Okayasu I. 2020. Positive correlation of expression of L-type amino-acid transporter 1 with colorectal tumor progression and prognosis: higher expression in sporadic colorectal tumors compared with ulcerative colitis-associated neoplasia. Pathol Res Pract. 216(6):152972. doi:10.1016/j.prp.2020.152972. 32359697

[jkad219-B87] Sawada S, Daimon H, Asakura S, Kawaguchi T, Yamatsu K, Furihata C, Matsushima T. 1994. Cumulative effects of chromosome aberrations and sister chromatid exchanges in rat liver induced in vivo by heterocyclic amines. Carcinogenesis. 15(2):285–290. doi:10.1093/carcin/15.2.285. 8313519

[jkad219-B88] Schürer KA, Rudolph C, Ulrich HD, Kramer W. 2004. Yeast *MPH1* gene functions in an error-free DNA damage bypass pathway that requires genes from homologous recombination, but not from postreplicative repair. Genetics. 166(4):1673–1686. doi:10.1534/genetics.166.4.1673. 15126389 PMC1470801

[jkad219-B89] Sengstag C, Weibel B, Fasullo M. 1996. Genotoxicity of aflatoxin B1: evidence for a recombination-mediated mechanism in *Saccharomyces cerevisiae*. Cancer Res. 56(23):5457–5465. 8968101 PMC10195021

[jkad219-B90] Shema E, Tirosh I, Aylon Y, Huang J, Ye C, Moskovits N, Raver-Shapira N, Minsky N, Pirngruber J, Tarcic G, et al 2017. Corrigendum: the histone H2B-specific ubiquitin ligase RNF20/hBRE1 acts as a putative tumor suppressor through selective regulation of gene expression. Genes Dev. 31(18):1926. doi:10.1101/gad.307207.117. 29051390 PMC5695091

[jkad219-B91] Sinha R, Chow WH, Kulldorff M, Denobile J, Butler J, Garcia-Closas M, Weil R, Hoover RN, Rothman N. 1999. Well-done, grilled red meat increases the risk of colorectal adenomas. Cancer Res. 59(17):4320–4324. 10485479

[jkad219-B92] Sinha R, Kulldorff M, Chow W-H, Denobile J, Rothman N. 2001. Dietary intake of heterocyclic amines, meat-derived mutagenic activity, and risk of colorectal adenomas. Cancer Epidemiol Biomarkers Prev. 10(5):559–562. 11352869

[jkad219-B93] Slattery ML, Potter JD, Bigler J, Caan B, Leppert M. 1998. *NAT2, GSTM-1,* cigarette smoking, and risk of colon cancer. Cancer Epidemiol Biomarkers Prev. 7(12):1079–1084. 9865425

[jkad219-B94] Smith AM, Heisler LE, Mellor J, Kaper F, Thompson MJ, Chee M, Roth FP, Giaever G, Nislow C. 2009. Quantitative phenotyping via deep barcode sequencing. Genome Res. 19(10):1836–1842. doi:10.1101/gr.093955.109. 19622793 PMC2765281

[jkad219-B95] Stavros KM, Hawkins EK, Rizzo CJ, Stone MP. 2014. Base-displaced intercalation of the 2-amino-3-methylimidazo[4,5-*f*]quinolone *N*^2^-dG adduct in the *Nar*I DNA recognition sequence. Nucleic Acids Res. 42(5):3450–3463. doi:10.1093/nar/gkt1109. 24366876 PMC3950664

[jkad219-B96] Sternberg PW, Agapite J, Albou LP, Aleksander SA, Alexander M, Anagnostopoulos AV, Antonazzo G, Argasinska J, Arnaboldi V, Attrill H. 2022. Alliance of Genome Resources Consortium. Harmonizing model organism data in the Alliance of Genome Resources. Genetics. 220(4):iyac022. doi:10.1093/genetics/iyac022. 35380658 PMC8982023

[jkad219-B97] St. John N, Freedland J, Baldino H, Doyle F, Cera C, Begley T, Fasullo M. 2020. Genome profiling for aflatoxin B1 resistance in *Saccharomyces cerevisiae* reveals a role for the CSM2/SHU Complex in tolerance of aflatoxin B1-associated DNA damage. G3 (Bethesda). 10(11):3929–3947. doi:10.1534/g3.120.401723. 32994210 PMC7642924

[jkad219-B98] Sun J, Nishiyama T, Shimizu K, Kadota K. 2013. TCC: an R package for comparing tag count data with robust normalization strategies. BMC Bioinformatics. 14(1):219. doi:10.1186/1471-2105-14-219. 23837715 PMC3716788

[jkad219-B99] Supek F, Bošnjak M, Škunca N, Šmuc T. 2011. Revigo summarizes and visualizes long lists of gene ontology terms. PLoS One. 6(7):e21800. doi:10.1371/journal.pone.0021800. 21789182 PMC3138752

[jkad219-B100] Szklarczyk D, Gable AL, Lyon D, Junge A, Wyder S, Huerta-Cepas J, Simonovic M, Doncheva NT, Morris JH, Bork P, et al 2019. STRING V11: protein-protein association networks with increased coverage, supporting functional discovery in genome-wide experimental datasets. Nucleic Acids Res. 47(D1):D607–D613. doi:10.1093/nar/gky1131. 30476243 PMC6323986

[jkad219-B101] Te Paske IBAW, Ligtenberg MJL, Hoogerbrugge N, de Voer RM. 2020. Candidate gene discovery in hereditary colorectal cancer and polyposis syndromes—considerations for future studies. Int J Mol Sci. 21(22):8757. doi:10.3390/ijms21228757. 33228212 PMC7699508

[jkad219-B102] Toczyski DP, Galgoczy DJ, Hartwell LH. 1997. *CDC5* And CKII control adaptation to the yeast DNA damage checkpoint. Cell. 90(6):1097–1106. doi:10.1016/S0092-8674(00)80375-X. 9323137

[jkad219-B103] Toussaint M, Levasseur G, Gervais-Bird J, Wellinger RJ, Elela SA, Conconi A. 2006. A high-throughput method to measure the sensitivity of yeast cells to genotoxic agents in liquid cultures. Mutat Res. 606(1–2):92–105. doi:10.1016/j.mrgentox.2006.03.006. 16713735

[jkad219-B104] Tsuji S, Kawasaki Y, Furukawa S, Taniue K, Hayashi T, Okuno M, Hiyoshi M, Kitayama J, Akiyama T. 2014. The miR-363-GATA6-Lgr5 pathway is critical for colorectal tumourigenesis. Nat Commun. 5:3150. doi:10.1038/ncomms4150. 24452072

[jkad219-B105] Turesky RJ, Gremaud E, Markovic J, Snyderwine EG. 1996. DNA Adduct formation of the food-derived mutagen 2-amino-3-methylimidazo[4,5-*f*]quinoline in nonhuman primates undergoing carcinogen bioassay. Chem Res Toxicol. 9(2):403–408. doi:10.1021/tx950132j. 8839042

[jkad219-B106] Turesky RJ, Le Marchand L. 2011. Metabolism and biomarkers of heterocyclic aromatic amines in molecular epidemiology studies: lessons learned from aromatic amines. Chem Res Toxicol. 24(8):1169–1214. doi:10.1021/tx200135s. 21688801 PMC3156293

[jkad219-B107] Turesky RJ, Markovic J. 1995. DNA Adduct formation of the food carcinogen 2-amino-3-methylimidazo[4, 5-f]quinoline (IQ) in liver, kidney and colo-rectum of rats. Carcinogenesis. 16(9):2275–2279. doi:10.1093/carcin/16.9.2275. 7554091

[jkad219-B108] Turesky RJ, Skipper PL, Tannenbaum SR. 1987. Binding of 2-amino-3-methylimidazo[4,5-f]quinoline to hemoglobin and albumin in vivo in the rat. Identification of an adduct suitable for dosimetry. Carcinogenesis. 8(10):1537–1542. doi:10.1093/carcin/8.10.1537. 3652389

[jkad219-B109] Turesky RJ, Vouros P. 2004. Formation and analysis of heterocyclic aromatic amine-DNA adducts in vitro and in vivo. J Chromatogr B Analyt Technol Biomed Life Sci. 802(1):155–166. doi:10.1016/j.jchromb.2003.10.053. 15036007

[jkad219-B110] Wang H, Yamamoto JF, Caberto C, Saltzman B, Decker R, Vogt TM, Yokochi L, Chanock S, Wilkens LR, Le Marchand L. 2011. Genetic variation in the bioactivation pathway for polycyclic hydrocarbons and heterocyclic amines in relation to risk of colorectal neoplasia. Carcinogenesis. 32(2):203–209. doi:10.1093/carcin/bgq237. 21081473 PMC3026844

[jkad219-B111] Warner JR . 1999. The economics of ribosome biosynthesis in yeast. Trends Biochem Sci. 24(11):437–440. doi:10.1016/S0968-0004(99)01460-7. 10542411

[jkad219-B112] Washington MT, Minko IG, Johnson RE, Haracska L, Harris TM, Lloyd RS, Prakash S, Prakash L. 2004. Efficient and error-free replication past a Minor-groove *N*^2^ -guanine adduct by the sequential action of yeast rev1 and DNA polymerase ζ. Mol Cell Biol. 24(16):6900–6906. doi:10.1128/mcb.24.16.6900-6906.2004. 15282292 PMC479736

[jkad219-B113] Weren RDA, Ligtenberg MJL, Kets CM, De Voer RM, Verwiel ETP, Spruijt L, Van Zelst-Stams WAG, Jongmans MC, Gilissen C, Hehir-Kwa JY, et al 2015. A germline homozygous mutation in the base-excision repair gene *NTHL1* causes adenomatous polyposis and colorectal cancer. Nat Genet. 47(6):668–671. doi:10.1038/ng.3287. 25938944

[jkad219-B114] Willander K, Falk IJ, Chaireti R, Paul E, Hermansson M, Gréen H, Lotfi K, Söderkvist P. 2014. Mutations in the isocitrate dehydrogenase 2 gene and *IDH1* SNP 105C > T have a prognostic value in acute myeloid leukemia. Biomark Res. 2(1):18. doi:10.1186/2050-7771-2-18. 25324972 PMC4198977

[jkad219-B115] Wooden SH, Bassett HM, Wood TG, McCullough AK. 2004. Identification of critical residues required for the mutation avoidance function of human MutY (hMYH) and implications in colorectal cancer. Cancer Lett. 205(1):89–95. doi:10.1016/j.canlet.2003.10.006. 15036665

[jkad219-B116] Wu HI, Brown JA, Dorie MJ, Lazzeroni L, Brown JM. 2004. Genome-wide identification of genes conferring resistance to the anticancer agents cisplatin, oxaliplatin, and mitomycin C. Cancer Res. 64(11):3940–3948. doi:10.1158/0008-5472.CAN-03-3113. 15173006

[jkad219-B117] Xu X, Blackwell S, Lin A, Li F, Qin Z, Xiao W. 2015. Error-free DNA-damage tolerance in *Saccharomyces cerevisiae*. Mutat Res Rev Mutat Res. 764:43–50. doi:10.1016/j.mrrev.2015.02.001. 26041265

[jkad219-B118] Yang Y, Gao Y, Zlatanou A, Tateishi S, Yurchenko V, Rogozin IB, Vaziri C. 2018. Diverse roles of RAD18 and Y-family DNA polymerases in tumorigenesis. Cell Cycle. 17(7):833–843. doi:10.1080/15384101.2018.1456296. 29683380 PMC6056224

[jkad219-B119] Yu J, Liu M, Liu H, Zhou L. 2019. GATA1 Promotes colorectal cancer cell proliferation, migration and invasion via activating AKT signaling pathway. Mol Cell Biochem. 457(1–2):191–199. doi:10.1007/s11010-019-03523-w. 31069596

[jkad219-B120] Yuen KWY, Warren CD, Chen O, Kwok T, Hieter P, Spencer FA. 2007. Systematic genome instability screens in yeast and their potential relevance to cancer. Proc Natl Acad Sci U S A. 104(10):3925–3930. doi:10.1073/pnas.0610642104. 17360454 PMC1820685

[jkad219-B121] Zhao Y, Ehara H, Akao Y, Shamoto M, Nakagawa Y, Banno Y, Deguchi T, Ohishi N, Yagi K, Nozawa Y. 2000. Increased activity and intranuclear expression of phospholipase D2 in human renal cancer. Biochem Biophys Res Commun. 278(1):140–143. doi:10.1006/bbrc.2000.3719. 11185526

